# A CDC42EP4/septin-based perisynaptic glial scaffold facilitates glutamate clearance

**DOI:** 10.1038/ncomms10090

**Published:** 2015-12-10

**Authors:** Natsumi Ageta-Ishihara, Maya Yamazaki, Kohtarou Konno, Hisako Nakayama, Manabu Abe, Kenji Hashimoto, Tomoki Nishioka, Kozo Kaibuchi, Satoko Hattori, Tsuyoshi Miyakawa, Kohichi Tanaka, Fathul Huda, Hirokazu Hirai, Kouichi Hashimoto, Masahiko Watanabe, Kenji Sakimura, Makoto Kinoshita

**Affiliations:** 1 Division of Biological Sciences, Department of Molecular Biology, Nagoya University Graduate School of Science, Nagoya 464-8602, Japan; 2 Department of Cellular Neurobiology, Brain Research Institute, Niigata University, Niigata 951-8585, Japan; 3 Department of Anatomy, Hokkaido University Graduate School of Medicine, Sapporo 060-8638, Japan; 4 Department of Neurophysiology, Graduate School of Biomedical and Health Sciences, Hiroshima University, Hiroshima 734-8551, Japan; 5 Division of Clinical Neuroscience, Chiba University Center for Forensic Mental Health, Chiba 260-8670, Japan; 6 Department of Cell Pharmacology, Nagoya University Graduate School of Medicine, Showa, Nagoya 466-8560, Japan; 7 Division of Systems Medical Science, Institute for Comprehensive Medical Science, Fujita Health University, Toyoake 470-1192, Japan; 8 Center for Genetic Analysis of Behavior, National Institute for Physiological Sciences, Okazaki 444-8585, Japan; 9 Laboratory of Molecular Neuroscience, Medical Research Institute, Tokyo Medical and Dental University, Tokyo 113-8510, Japan; 10 Department of Neurophysiology and Neural Repair, Gunma University Graduate School of Medicine, Maebashi, Gunma 371-8511, Japan

## Abstract

The small GTPase-effector proteins CDC42EP1-5/BORG1–5 interact reciprocally with CDC42 or the septin cytoskeleton. Here we show that, in the cerebellum, CDC42EP4 is exclusively expressed in Bergmann glia and localizes beneath specific membrane domains enwrapping dendritic spines of Purkinje cells. CDC42EP4 forms complexes with septin hetero-oligomers, which interact with a subset of glutamate transporter GLAST/EAAT1. In *Cdc42ep4*^−/−^ mice, GLAST is dissociated from septins and is delocalized away from the parallel fibre-Purkinje cell synapses. The excitatory postsynaptic current exhibits a protracted decay time constant, reduced sensitivity to a competitive inhibitor of the AMPA-type glutamate receptors (γDGG) and excessive baseline inward current in response to a subthreshold dose of a nonselective inhibitor of the glutamate transporters/EAAT1–5 (DL-TBOA). Insufficient glutamate-buffering/clearance capacity in these mice manifests as motor coordination/learning defects, which are aggravated with subthreshold DL-TBOA. We propose that the CDC42EP4/septin-based glial scaffold facilitates perisynaptic localization of GLAST and optimizes the efficiency of glutamate-buffering and clearance.

## Abstract (web summary)

Glutamate transporters mediate neurotransmitter reuptake at glutamatergic synapses. Here the authors show that CDC42 effector protein CDC42EP4 supports efficient glutamate clearance by promoting the tethering of a glutamate transporter GLAST to perisynaptic clusters of septins in Bergmann glia.

## Graphical abstract

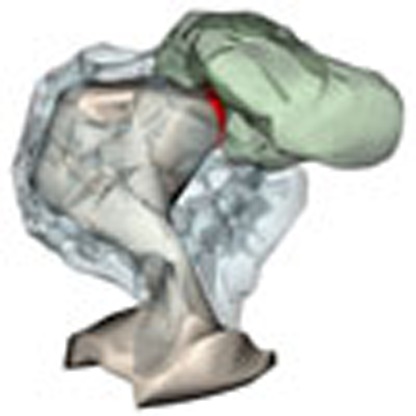

Glutamate transporters (Excitatory Amino Acid Transporter 1–5/EAAT1–5) are membrane-bound solute carrier proteins that terminate glutamatergic neurotransmission and maintain the glutamate homeostasis by the symport of extracellular glutamate and Na^+^/H^+^ into the glial and neuronal cytoplasm^1^. The extracellular glutamate concentration surges to 160–250 μM near perisynaptic domains of Bergmann glia in the cerebellum, which is rapidly buffered and cleared by binding to and reuptake through EAATs^2^,^3^. When the glutamate-buffering/clearance capacity falls short of glutamate-release activities, glutamate pervades the extracellular space. Excessive external glutamate entails protracted activation of glutamate receptors in the nearest synaptic and extrasynaptic membranes, and in the neighbouring synapses, and of glial responses. In the cerebellar molecular layer, GLAST/EAAT1/SLC1A3 is highly concentrated along perisynaptic Bergmann glial membranes^4^ and plays a major role in the clearance of glutamate released from the parallel fibres (PFs, from granule cells in the cerebellar granule cell layer) and climbing fibres (CFs, from neurons in the inferior olivary nuclei) to Purkinje cells (PCs), while GLT-1/EAAT2, the major glial transporter in the forebrain, and neuronal EAAT4 play relatively minor roles^5^. Genetic loss of GLAST results in anomalous CF–PC innervations and defective motor coordination/learning^6^, attesting its central role in Bergmann glia-mediated glutamate homeostasis in the cerebellum.

Despite the developmental and physiological significance of GLAST, and the linkage to human episodic ataxia and schizophrenia^7^,^8^,^9^, little is known about the post-translational regulation. Physical interactions with beta-III spectrin/GTRAP41 and ARHGEF11/GTRAP48 were reported to facilitate the anchorage and activity of EAAT4 (ref. 10). However, physiological significance of these findings from heterologous cells, and whether these proteins modulate the localization and/or activity of GLAST, remains unknown. Previous studies demonstrated a physical interaction between GLAST and septins (a family of polymerizing GTPases that constitute the membrane skeleton) *in vitro* and in heterologous cells, and their partial co-localization in Bergmann glia^11^,^12^. However, hypothetical septin dependence of the perisynaptic targeting and activity of GLAST has never been directly tested *in vivo*, partly because of the redundancy among the septin family (see Discussion).

Another line of biochemical and cell biological studies showed that a family of CRIB-domain proteins CDC42EPs/BORGs binds to septin hetero-oligomers or CDC42 (a signalling small GTPase that controls cytoskeleton and cell morphogenesis) in a mutually exclusive manner. The major determinant of the binding preference of CDC42EPs is the status of CDC42-bound nucleotide; GTP-CDC42>septins>GDP-CDC42 (refs 13, 14, 15). However, again, *in vivo* relevance of the hypothetical CDC42-CDC42EP-septin pathway remains unclear.

Given the above background, genetic deletion of the dominant CDC42EP species in Bergmann glia is a rational approach to address the two open issues: physiological roles of the CDC42-CDC42EP-septin pathway and of the septin–GLAST interaction in the brain. We find robust co-expression and co-localization of CDC42EP4 and septins in Bergmann glia, generate CDC42EP4-null mice and conduct biochemical, fine morphological, electrophysiological, pharmacological and behavioural analyses. The unique and systematic approach reveals the requirement of CDC42EP4 in Bergmann glia for the septin-mediated perisynaptic localization of GLAST, and for the efficient buffering and clearance of glutamate from around synapses towards PCs.

## Results

### Selective expression of CDC42EP4 in Bergmann glia

**Figure 1:**
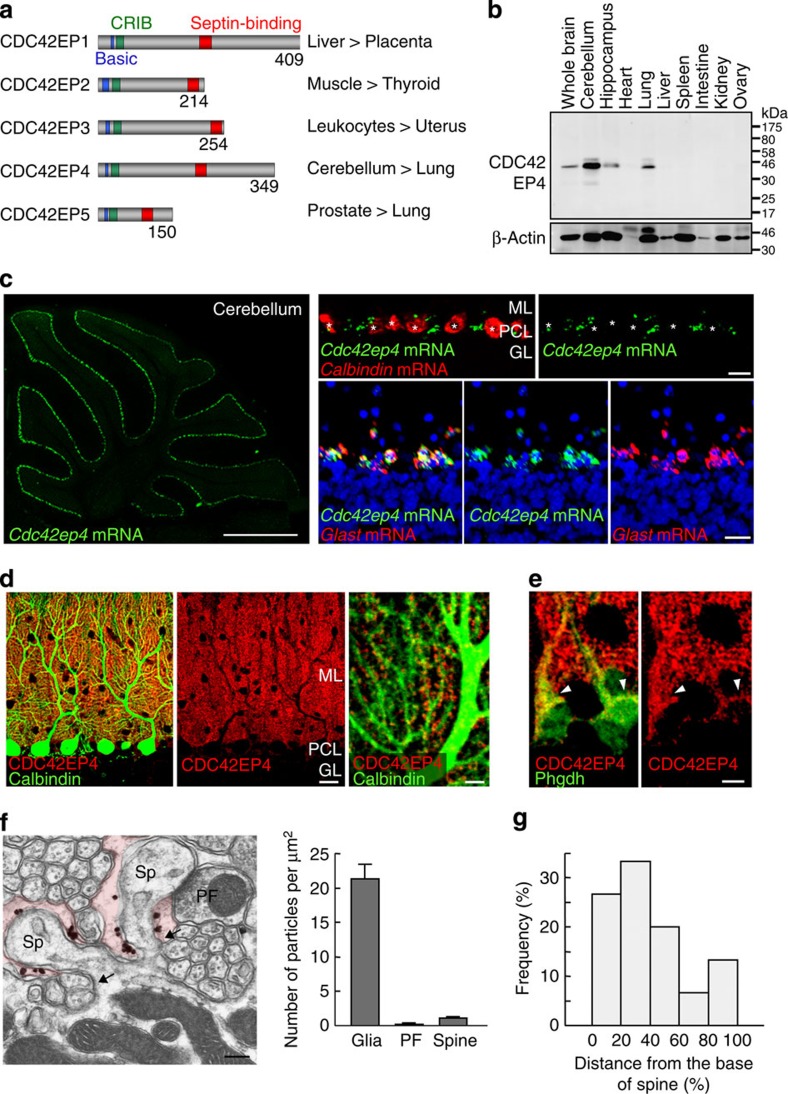
Bergmann glia-selective expression and unique perisynaptic localization of CDC42EP4. (**a**) The CDC42EP/BORG family and gene expression pattern in the mouse. Each CDC42EP contains a set of basic-CRIB-BD domains. Anti-CDC42EP4 antibody was raised against a region between the CRIB and BD3 (septin-binding) domains. The numbers denote amino-acid residues. CDC42EP1/2/3/4/5, respectively, corresponds to BORG5/1/2/4/3. (**b**) Lysates from adult mouse tissues (50 μg total protein per lane) were immunoblotted for CDC42EP4 and were reprobed for β-actin as a loading control. The major ∼39-kDa band was most abundant in the cerebellum. (**c**) FISH for *Cdc42ep4* mRNA in the adult mouse cerebellum. (Left) Labels for *Cdc42ep4* (green) highlighted the PC layer in a parasagittal section. (Top) Double-label FISH for mRNAs for *Cdc42ep4* and a PC marker calbindin (red). *Cdc42ep4* mRNA was excluded from PCs (*). (Bottom) Double-label FISH for *Cdc42ep4* and *Glast* (red) mRNAs, and TOTO-3 stain for DNA (blue). The two mRNA signals overlapped in all (*n*=115) Bergmann glial cells identified in a representative section. Scale bars, 1mm and 20 μm. (**d**) (Left and centre) Double-label IF for CDC42EP4 (red) and calbindin (green) in the cerebellar cortex. The diffuse granular signals for CDC42EP4 distributed throughout the molecular layer and in the PC layer, which were excluded from PCs and the granule cell layer. (Right) At a higher magnification, CDC42EP4-positive puncta were interspersed and aligned along PC dendrites. Scale bars, 20 and 5 μm. (**e**) Double-label IF for CDC42EP4 (red) and a Bergmann glial marker Phgdh (green). The limited overlap in Bergmann glial cell bodies (arrowheads) indicated selective localization of CDC42EP4 in glial processes. Scale bar, 5 μm. (**f**) (Left) Immunoelectron microscopy image for CDC42EP4 in the molecular layer. Gold particles for CDC42EP4 were found as submembranous clusters in terminal processes of Bergmann glia (tinted), each surrounding a dendritic spine (Sp) of a PC. PF, parallel fibre terminal. Scale bar, 200 nm. (Right) Quantification of glia-selective localization of CDC42EP4. Data represented as mean±s.e.m. (**g**) Histogram showing a gradient of CDC42EP4 signals relative to the geometry of dendritic spines of PCs; higher in regions facing spine base (arrows in **f**) than in regions facing the spine head.

We conducted immunoblot (IB) for the expression profiling of the CDC42EP4 protein in the whole brain, two brain subregions (cerebellum and hippocampus) and seven non-neural tissues from adult C57BL/6N mice (Fig. 1a). The major band of ∼39 kDa, which fits the calculated molecular mass of 37,980, was the most abundant in the cerebellum (Fig. 1b and Supplementary Fig. 15) as predicted from public gene expression databases including National Center for Biotechnology Information.

Fluorescence *in situ* hybridization (FISH) for *Cdc42ep4* mRNA highlighted the PC layer in the cerebellum (Fig. 1c, left), which is consistent with the data in the Allen Mouse Brain Atlas #71723875 (Allen Institute for Brain Science). Double-label FISH showed complementary distribution of mRNAs for CDC42EP4 and calbindin (PC marker) and overlap between mRNAs for CDC42EP4 and GLAST (Bergmann glia marker; Fig. 1c, right), demonstrating highly Bergmann glia-selective expression of the *Cdc42ep4* gene.

### Clustering of CDC42EP4 in perisynaptic glial processes

Consistently, double-label immunofluorescence (IF) for CDC42EP4 and calbindin, respectively, highlighted Bergmann glia and PCs in a mutually exclusive manner (Fig. 1d). At a higher magnification with a lower gain level, CDC42EP4 immunoreactivity appeared as ‘hotspots', which were interspersed along PC dendrites and tightly apposed to dendritic spines (Fig. 1d, right). CDC42EP4 was far more concentrated in the processes of Bergmann glia than in the cell bodies (Fig. 1e).

To analyse the subcellular distribution of CDC42EP4 at higher resolutions, we conducted silver-enhanced immunoelectron microscopy. Bergmann glial processes thoroughly ensheathed dendritic spines of PCs, except for synaptic contact sites with axon terminals. Gold particles for CDC42EP4 were commonly found as submembranous clusters in Bergmann glial processes that surrounded dendritic spines, particularly around the spine neck (Fig. 1f). Quantitative analysis demonstrated a gradient of CDC42EP4 from the base (neck) to the apex (head) of spines (Fig. 1g). These data indicate that CDC42EP4 in Bergmann glia is localized beneath specific membrane domains that are facing dendritic spines of PCs. The characteristic distribution of CDC42EP4 is reminiscent of those of septin subunits, SEPT4/H5 (ref. 16), SEPT7/hCDC10 (ref. 16) and SEPT2/Nedd5 (ref. 11), whose physiological role has been unknown.

### Generation of *Cdc42ep4*-floxed and -null mice

**Figure 2:**
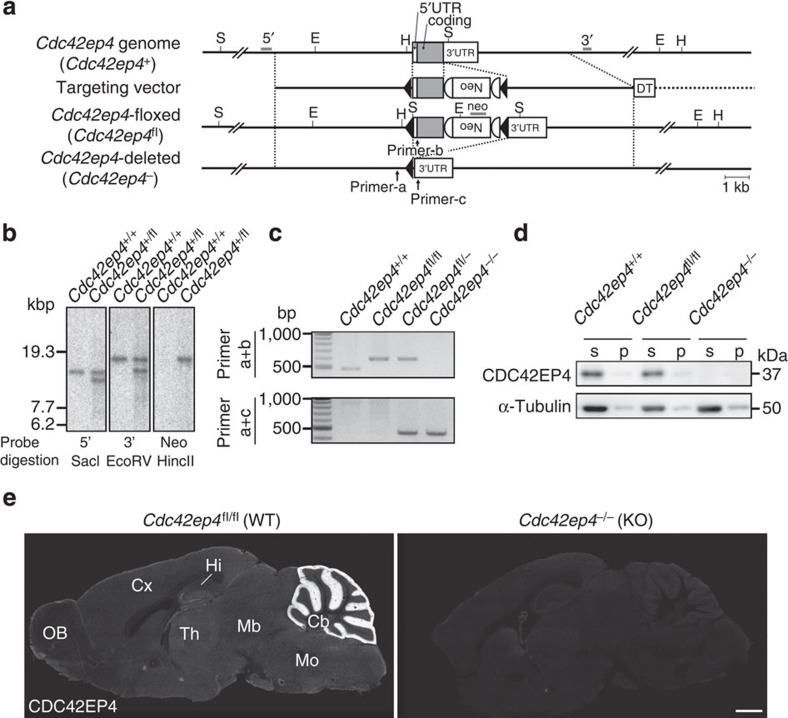
Generation of *Cdc42ep4*^−/−^ mice and morphology of the major neuronal components. (**a**) The KO strategy of the *Cdc42ep4* gene. A schematic diagram showing the wild-type (*Cdc42ep4*^+^), floxed (*Cdc42ep4*^fl^) and null (*Cdc42ep4*^−^) alleles, and the targeting vector. Note that Cre-mediated loxP recombination leaves no coding exon. The restriction sites for Sac I (S), EcoR V (E) and Hinc II (H), three probes (grey bars) used for Southern blot analysis and three PCR primer sites are indicated. Signs; coding region (grey box), untranslated region (open box), loxP (black triangle), frt (open half-circle), neomycin resistance cassette (Neo), diphtheria toxin A-chain cassette (DT). (**b**) Southern blot analysis. Genomic DNAs purified from WT and the chimera (*Cdc42ep4*^+/+^;*Cdc42ep4*^fl/+^) mice were digested with the restriction enzymes and hybridized with the probes as indicated. The band patterns, as seen in preceding Southern blot analysis of ES cell clones, reconfirmed successful homologous recombination of the clone. See Methods for details. (**c**) PCR genotyping. Two sets of primers discriminated genomic DNAs from *Cdc42ep4*^+/+^, *Cdc42ep4*^fl/fl^ (WT), *Cdc42ep4*^fl/−^ and *Cdc42ep4*^−/−^ (KO) mice. (**d**) Expression and extractability of CDC42EP4 in the adult mouse cerebellum. The pellet/supernatant assay showed that CDC42EP4 is partitioned mostly to the detergent-extractable, supernatant (s) fraction but not to the inextractable, pellet (p) fraction. CDC42EP4 was absent from KO tissues. α-Tubulin was used as a loading control. (**e**) IF for CDC42EP4 on parasagittal brain sections of adult male littermate WT and KO mice. The molecular layer of the WT cerebellum (Cb) was intensely labelled for CDC42EP4, which was absent from the KO brain. The faint, diffuse labelling of the entire brain is attributed to astrocytes. These results are consistent with the immunoblot data (Figs 1b and 2d) and warrant the specificity (high signal-to-noise ratio) of the antibody. Scale bar, 1 mm. UTR, untranslated repeat.

To explore physiological roles of *Cdc42ep4* using a reverse genetic approach, we generated a line of C57BL/6N mice harbouring a floxed allele, which was subsequently converted to a null allele by crossing with another line that ubiquitously expresses Cre recombinase (Fig. 2a–c). Analyses of *Cdc42ep4*^−/−^ offspring demonstrated the total absence of CDC42EP4 in IB and IF (Fig. 2d,e and Supplementary Fig. 15). For the following analyses, we consistently compared *Cdc42ep4*^−/−^ (knockout; KO) and *Cdc42ep4*^fl/fl^ (wild type; WT) male littermates generated from *Cdc42ep4*^fl/−^ (heterozygous) parents. There was no difference by genotype in the gross appearance, body weight and fertility (Supplementary Fig. 1).

### Normal architecture of the *Cdc42ep4* ^*−/−*^ cerebellum

**Figure 3:**
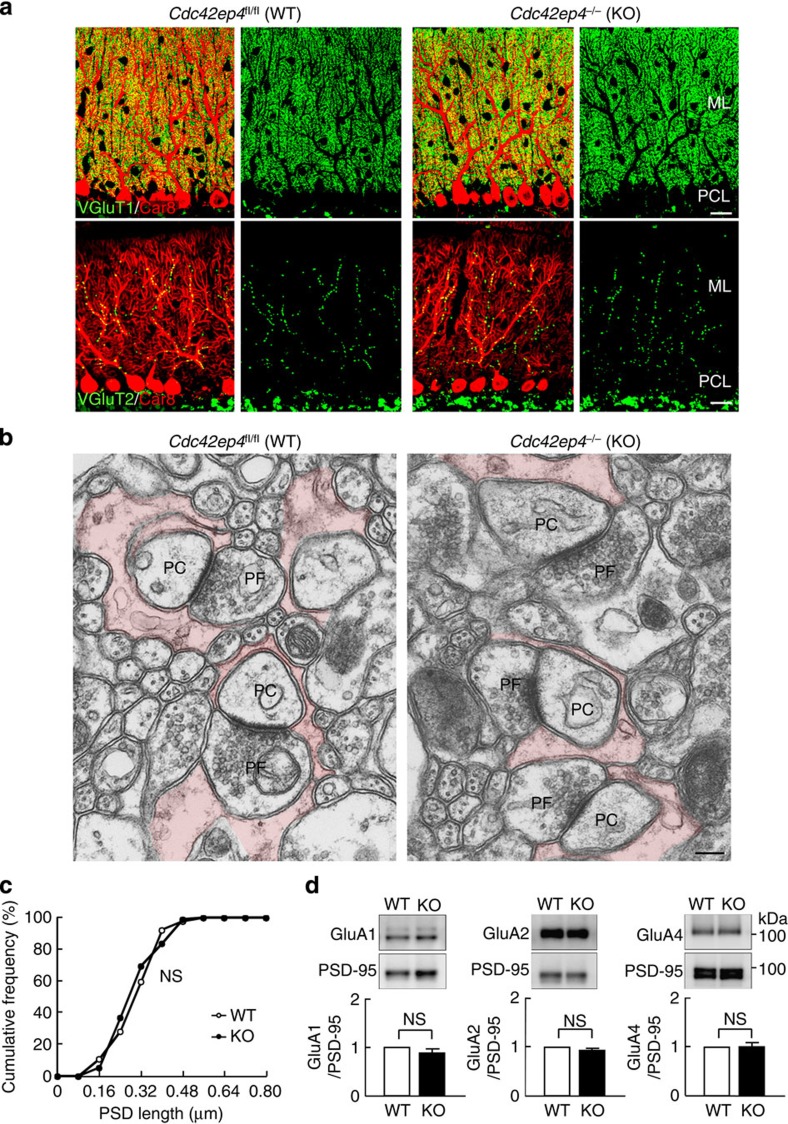
Morphological analysis of the neuronal and glial components in *Cdc42ep4*^fl/fl^ and *Cdc42ep4*^−/−^ cerebellar cortices. (**a**) Double-label IF of WT and KO cerebellar cortices for a Purkinje cell marker Car8 (red) and a parallel fibre (that is, granule cell) marker VGluT1 (top, green) or a climbing fibre marker VGluT2 (bottom, green). No obvious morphological anomaly, including aberrant CF–PC innervation, was found in the major neuronal components of KO-derived samples. Scale bar, 20 μm. (**b**) Transmission electron microscopy images of WT and KO molecular layers. No obvious ultrastructural difference was found between the genotypes. PF, parallel fibre terminal or bouton. PC, dendritic spine of Purkinje cell. Bergmann glial processes are tinted. Scale bar, 200 nm. (**c**) Cumulative histogram of PSD length of the PF–PC synapses, showing no significant difference between the genotypes (*n*=92 synapses from two littermates for each genotype, NS, *P*>0.05 by Kolmogorov–Smirnov test). (**d**) Quantitative immunoblot of WT and KO cerebellar PSD fractions for GluA1, GluA2 and GluA4 (the major subunits of the AMPARs), each normalized with PSD-95. There was no significant quantitative difference by genotype (*n*=3, NS, *P*>0.05 by *t*-test).

We conducted IF with specific markers for the four major neuronal and glial components (PFs, CFs, PCs and Bergmann glia), which did not show obvious anomaly in the cerebellar cortex of KO mice (Figs 3a and 5b). Transmission electron microscopy (TEM) images showed no recognizable ultrastructural defects in the molecular layer (Fig. 3b), including the lengths of the postsynaptic density (PSD) in PF–PC synapses (Fig. 3c). Four major proteins at glutamatergic synapses, GluA1, GluA2, GluA4 and PSD-95, did not show quantitative differences by genotype (Fig. 3d and Supplementary Fig. 15). These data indicate that CDC42EP4 is dispensable for the morphological development of the major neuronal and glial components, and synapse architecture in the cerebellar cortex.

### Septin oligomers as the major binding partners of CDC42EP4

For unbiased identification of physiological binding partners of CDC42EP4 in Bergmann glia, we conducted proteomic analysis of cerebellar lysates. Immunoaffinity chromatography for CDC42EP4 followed by mass spectrometry identified CDC42EP4, nine septin subunits (SEPT2/3/4/5/6/7/8/10/11) and a few other cytoskeletal proteins including Myosin-10 (nonmuscle myosin heavy chain IIB) and α-II/β-II spectrins (Table 1). The minimal background noises in the three negative control experiments (for example, anti-CDC42EP4 IgG captured no peptides from KO samples) reconfirmed the absence of CDC42EP4 in the KO cerebellum and the antibody specificity. Intriguingly, however, CDC42 and Tc10/RhoQ (another small GTPase that can bind to CDC42EPs *in vitro*^13^) were not detected (Table 1 and Supplementary Fig. 13). Thus, the major physiological binding partners of CDC42EP4 in the adult mouse cerebellum are not small GTPases, but hetero-oligomers of septins.

### Physiological network of CDC42EP4, septins and GLAST

**Figure 4:**
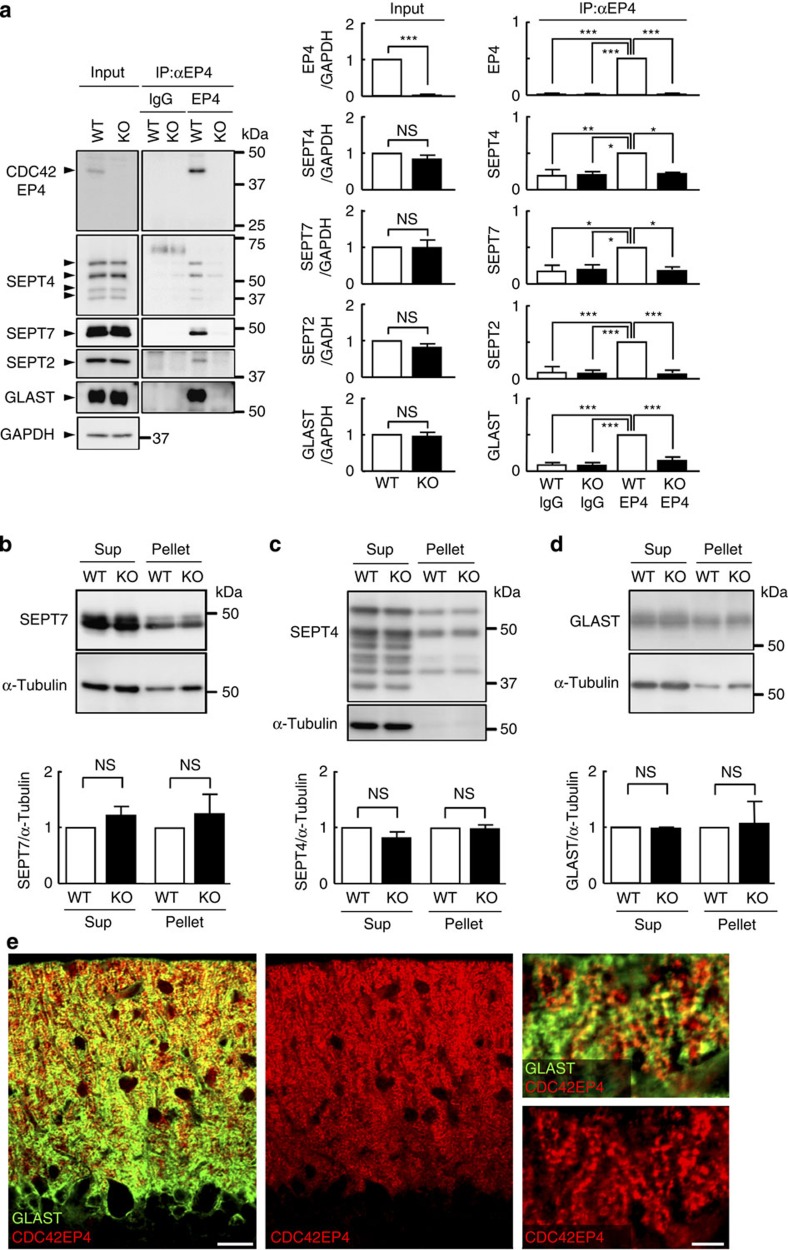
Biochemical analysis of binding partners of CDC42EP4 in *Cdc42ep4*^fl/fl^ and *Cdc42ep4*^−/−^ cerebella. (**a**) Co-IP/IB assay of CDC42EP4 with representative septin subunits and GLAST from WT and KO cerebellar lysates. (Input) IB for SEPT4, SEPT7, SEPT2 and GLAST, respectively, detected a quadruplet of 54, 52, 48 and 44 kDa, a doublet of 51 and 48 kDa, a single 42 kDa band and a broad 55 kDa band in the cerebellar lysate. (IP) Anti-CDC42EP4 antibody pulled down SEPT4, SEPT7, SEPT2 and GLAST only from WT cerebellar lysate. The graphs show densitometric quantification of the yield (*n*=3, ***P<0.001, **P<0.01, *P<0.05, NS, *P*>0.05 by one-way ANOVA with *post hoc* Tukey test). (Note: the extraction condition including the lysis buffer composition was optimized to detect GLAST, which was distinct from the one used mainly for the proteomic analysis (Table 1). See Methods.) (**b**–**d**) Pellet/supernatant assay results on the quantity and extractability of SEPT7, SEPT4 and GLAST in WT and KO cerebella. There was no significant difference in their amount and partitioning by genotype (*n*=3, NS, *P*>0.05 by *t*-test). The same membranes were reprobed for α-tubulin as a loading control, which was used for normalization. (**e**) Double-label IF for GLAST (green) and CDC42EP4 (red) in WT cerebellar cortex showing their partial co-localization in Bergmann glial processes. Scale bars, 20 and 5 μm.

Previous studies with recombinant proteins separately demonstrated direct interactions between CDC42EP5/BORG3 and septin hetero-oligomers (for example, SEPT6/7 and SEPT2/6/7, but not individual subunits)^14^,^15^, and between SEPT2 and GLAST^11^. However, higher-order molecular network composed of CDC42EPs, septin hetero-oligomers and GLAST has never been tested. We addressed this with their co-immunoprecipitation (co-IP) from cerebellar lysate (Fig. 4a and Supplementary Figs 13–15), and double-label IF that showed their partial overlap in Bergmann glia (Fig. 4e). These data, along with the previous studies and our proteomic and IF findings, indicate physiological molecular network that contains CDC42EP4, septin hetero-oligomers and a subset of GLAST.

### Loss of CDC42EP4 diminishes septin–GLAST interaction

**Figure 5:**
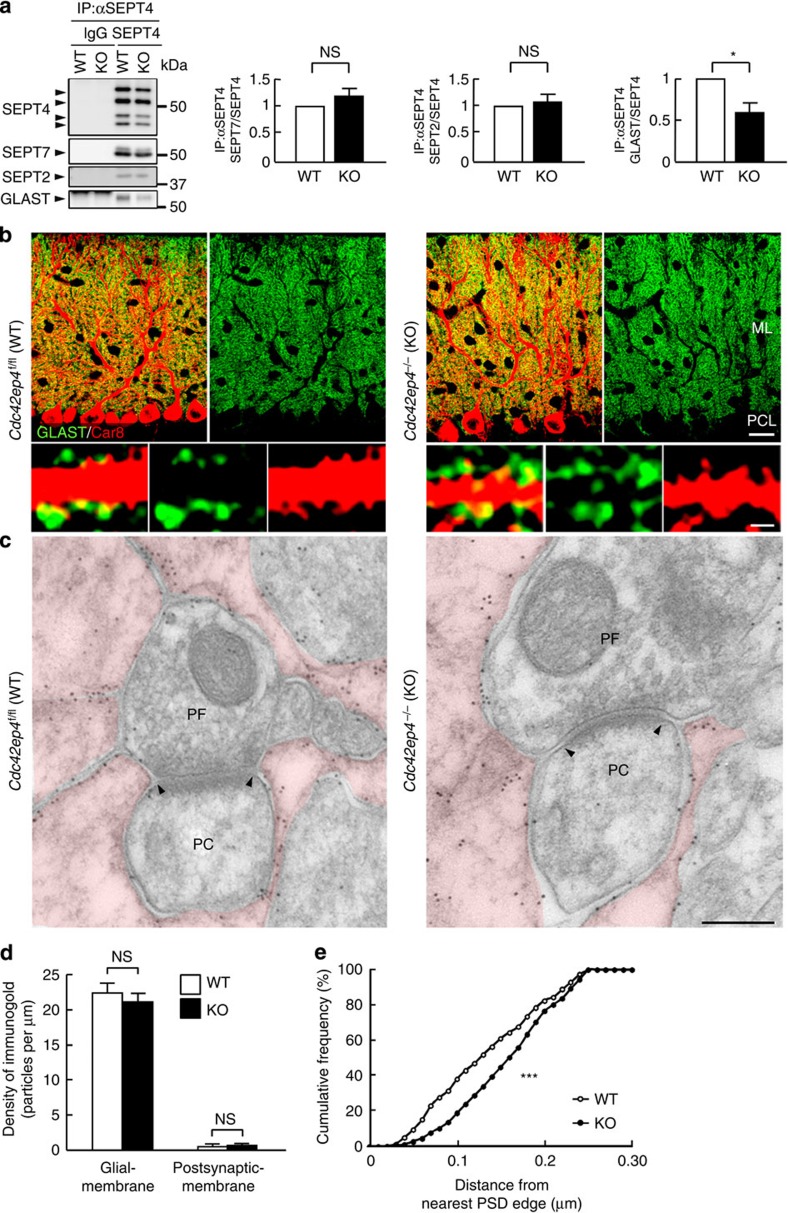
Loss of CDC42EP4 dissociates the GLAST–septin interaction and delocalizes GLAST away from synapses. (**a**) Quantitative co-IP/IB assay between SEPT4 and SEPT7, SEPT2 or GLAST in WT and KO cerebellar lysates. While the interaction among the septin subunits did not differ, the relative amount of GLAST pulled down with SEPT4 from KO lysate was significantly less than that from WT (*n*=3, *P<0.05, NS, *P*>0.05 by *t*-test), indicating that septin–GLAST interaction depends on CDC42EP4. See Fig. 4a–d for the comparable amount and solubility of these proteins in WT and KO cerebella. (**b**) Double-label IF for GLAST (green) and a Purkinje cell marker Car8 (red) in WT and KO cerebellar cortices. Genetic loss of CDC42EP4 caused no recognizable difference in the distribution of GLAST up to the resolution. Scale bars, 20 and 1 μm. (**c**) Immunoelectron microscopy images for GLAST in WT and KO molecular layers. PF, parallel fibre terminal or bouton. PC, dendritic spine of Purkinje cell. Bergmann glial processes are tinted. The pattern of GLAST distribution appears comparable to that of a previous study^4^. Scale bar, 200 nm. (**d**) Quantitative analysis of immunoelectron microscopy data. Bergmann glia selectivity and labelling density of GLAST were comparable between WT and KO mice: Bergmann glia; 22.4±1.3/21.1±1.2 particles per μm (*n*=519/467 particles from two littermate pairs, NS, *P*>0.05 by Mann–Whitney *U*-test). Postsynaptic membrane; 0.50±0.21/0.68±0.24 particles per μm (*n*=5/8 particles, *P*=0.68 by Mann–Whitney *U*-test; *cf*. Figs 4d and 5a–c). (**e**) Cumulative histogram of the distance of GLAST measured from the nearest PSD edge (for example, arrowheads in **c**). A significant right shift of the curve for KO mice demonstrates delocalization of GLAST away from PSDs of PF–PC synapses (median, WT=0.27 μm, KO=0.31 μm from the nearest edge of PSD; *n*=22/19 synapses from two littermates for each genotype, ***P<0.001 by Kolmogorov–Smirnov test).

To validate the significance of CDC42EP4 in the molecular network, we compared the status with or without CDC42EP4. Pellet/supernatant assay of cerebellar lysates showed that the amount and solubility of SEPT7, SEPT4 and GLAST were unaffected by the loss of CDC42EP4 (Fig. 4b–d and Supplementary Fig. 15). Their distribution patterns assessed using IF showed no recognizable difference by genotype (Fig. 5b). Intriguingly, however, a co-IP/IB assay revealed a significant reduction (Δ50%) of GLAST that was pulled down with SEPT4 (a Bergmann glia-selective septin subunit) from the *Cdc42ep4*^−/−^ cerebellum, when the interactions with two other major septin subunits in Bergmann glia (SEPT2 and SEPT7) were unaffected (Fig. 5a and Supplementary Fig. 15). The significant dissociation of GLAST from septin hetero-oligomers by the loss of CDC42EP4 indicates a role for CDC42EP4 as a stabilizer and/or an adapter for the association between GLAST and septin hetero-oligomers.

### GLAST delocalizes away from perisynapse without CDC42EP4

Previous fluorescence recovery after photobleaching assay showed that septin depletion via RNA interference augmented diffusional mobility of green fluorescent protein-tagged GLAST on the plasma membrane, while septin filament stabilization gave the opposite effect, indicating a role for septins as submembranous scaffold and/or diffusion barrier for GLAST^12^. To assess whether the dissociation from septins by the loss of CDC42EP4 could alter the distribution of GLAST, we conducted quantitative mapping of GLAST in Bergmann glia by postembedding immunoelectron microscopy technique (Fig. 5c–e). As expected, gold particles for GLAST were distributed along Bergmann glia membrane enwrapping pre- and postsynaptic elements of PF–PC synapses, whereas neuronal membranes were scarcely labelled. The distribution and density of immunogold particles were comparable between WT and KO mice (Fig. 5d). Intriguingly, however, the distance from perisynaptic GLAST signals to the nearest PSD edge (arrowheads, Fig. 5c) was significantly longer in KO mice than that in WT mice (Fig. 5e). Together, the biochemical and fine morphometric data consistently indicate that CDC42EP4 in Bergmann glia is required for GLAST to interact with septins and to localize along perisynaptic membrane domains enwrapping PF–PC synapses.

### *Cdc42ep4* ^*−/−*^ mice exhibit insufficient glutamate clearance

**Figure 6:**
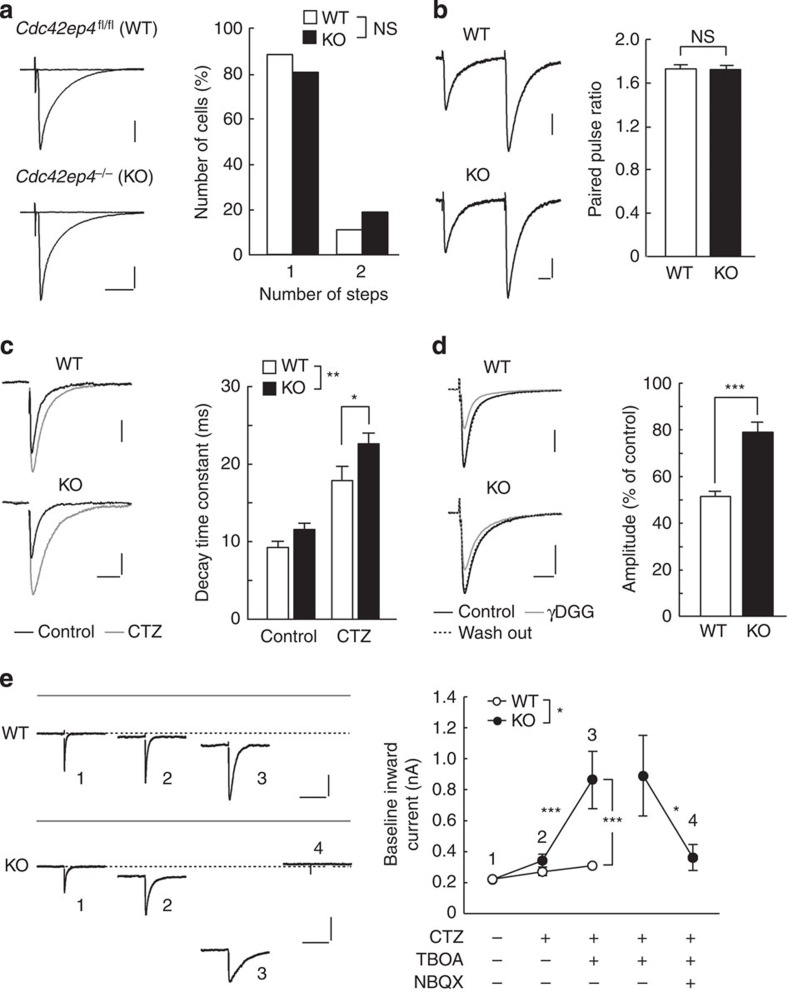
*Cdc42ep4*^−/−^ mice exhibit insufficient glutamate-buffering/clearance capacity. (**a**; Left) Sample traces of CF-EPSCs. Two to three traces were superimposed. Scale bars, 10 ms and 500 pA. (Right) Summary histogram showing the number of CF-EPSC steps (*n*=21/18, *P*=0.513 by Mann–Whitney *U*-test). Holding potential was −10 mV in **a** and −70 mV in **b**–**e**. (**b**; Left) PF-EPSCs in response to paired stimuli at 50 ms intervals. Scale bars, 10 ms and 100 pA. (Right) Summary graph of the paired-pulse ratio (*n*=22/19, *P*=0.824 by Mann–Whitney *U*-test). (**c**; Left) Sample traces of PF-EPSCs with (grey) or without (black) 100 μM CTZ in a WT and a KO mice. Scale bars, 25 ms and 50 pA. (Right) The decay time constant of PF-EPSCs. Although CTZ prolonged the decay time constant both in WT and KO (*n*=11/17, WT, from 9.3±0.8 to 17.9±1.8 ms, ***P<0.001; KO, from 11.6±0.8 to 22.6±1.3 ms; ***P<0.001), the effect was significantly larger in KO than in WT (**P=0.006 by two-way ANOVA with *post hoc* Tukey test), which resulted in more protracted postsynaptic response in KO PCs (*P=0.010). (**d**) (Left) PF-EPSCs before (black), in the presence of 1 mM γDGG (grey), and after washout (dashed). Scale bars, 10 ms and 200 pA. (Right) Summary histogram showing the effects of γDGG. KO PF-EPSCs were significantly more insensitive to γDGG than WT ones (*n*=7/7, ***P<0.001 by Mann–Whitney *U*-test). (**e**; Left) Sample traces of PF-EPSCs in a WT and a KO mice in control ACSF (1), in the presence of CTZ (2), CTZ plus 50 μM TBOA (3), CTZ, TBOA plus 10 μM NBQX (4). Grey and dashed lines, respectively, indicate the zero offset level and baseline holding current level in the control ACSF. Scale bars, 200 ms and 100 pA. (Right) The summary graph showing the holding current. The effects of CTZ plus TBOA was significantly larger on KO than on WT (*n*=11/12, ***P<0.001 by two-way repeated measures ANOVA with *post hoc* Tukey test). (Two right plots) Additional application of NBQX significantly diminished the inward current by TBOA (*n*=7, *P=0.016 by Wilcoxon signed-rank test).

To explore whether the anomalies of GLAST (that is, dissociation from septins and delocalization from synapses) in *Cdc42ep4*^−/−^ mice affect glutamatergic neurotransmissions, we assessed electrophysiological properties of the major glutamatergic synapses in cerebellar slices. We first examined the CF–PC synapse by stimulating CFs in the granule cell layer and recording the evoked excitatory postsynaptic currents (EPSCs) with a whole-cell voltage clamp configuration (Fig. 6a). Most PCs in 7-week-old WT (*n*=16/18, 89%) and KO (*n*=17/21, 81%) mice elicited a single large EPSC in an all-or-none manner as stimulus intensity gradually increased (Fig. 6a, left). The number of steps, which reflects the number of CFs innervating a given PC^17^,^18^, was comparable between WT and KO mice (Fig. 6a, right), indicating that CDC42EP4 is dispensable for the postnatal establishment of CF–PC monoinnervation. The major parameters of CF-EPSC kinetics (that is, amplitude, 10–90% rise time and decay time constant) were also comparable (Supplementary Table 1). These data indicate that properties of the CF-mediated synaptic transmission are normal in young adult KO mice.

Given the major role for GLAST in glutamate clearance from extracellular space around PF–PC synapses^5^, the delocalization of GLAST away from PSD (Fig. 5e) might affect PF–EPSC kinetics in KO mice. To test this, we measured the decay time constant of PF-EPSC, which is known to protract as glutamate reuptake delays^19^,^20^,^21^,^22^. The PF-EPSCs elicited by the stimulation in the molecular layer of KO mice showed normal paired-pulse facilitation (Fig. 6b and Supplementary Table 1), indicating that the presynaptic functions were largely unaffected. As previous studies showed that desensitization of the AMPA receptors (AMPARs) masks the effects of GLAST insufficiency on PF–PC synapses^20^, we also employed cyclothiazide (CTZ) to block desensitization of AMPARs. While bath application of CTZ prolonged the decay time constant of PF-EPSCs in both genotypes, it was significantly longer in KO than in WT (*P*=0.010, Fig. 6c). These data suggest a recognizable delay in the glutamate clearance from PF–PC synapses in KO mice.

To corroborate the above findings, we compared glutamate transients in PF-PC synapses using a low-affinity competitive inhibitor of the AMPARs, γ-D-glutamylglycine (γDGG). γDGG is a rapidly unbinding competitive antagonist that is readily displaced from AMPARs by released glutamate^23^. Therefore, the degree of inhibition of the EPSC roughly reflects the relative size and kinetics of the glutamate transient in the synaptic cleft. Bath application of 1 mM γDGG inhibited the PF-EPSC amplitude to ∼50% of the basal level in WT, while only to ∼80% in KO, suggesting that the size of glutamate transient at PF–PC synapses was significantly higher in KO than in WT (WT versus KO, 51.5±2.2/79.1±4.2%, *n*=7, *P*<0.001, Fig. 6d).

The above findings, in conjunction with the molecular-level anomalies, suggest that PF–PC synapses in KO mice have hotspots around active zones with subnormal glutamate-buffering capacity. Since the density of AMPARs is the highest in postsynaptic active zones, we hypothesized that KO mice are sensitive to subthreshold doses of glutamate clearance inhibitors. To test this, we applied 50 μM DL-TBOA, which nonselectively inhibits glutamate transporters (EAAT1–5) but only partially as the half-maximal inhibitory concentration for EAAT1/GLAST is 70 μM. The treatment with the low-dose DL-TBOA in addition to CTZ elicited a significantly larger baseline inward current in KO PCs (WT versus KO, 310±26/864±185 pA, *n*=11/12, *P*<0.001, Fig. 6e, trace 3). The aberrant baseline inward current of KO mice was abolished by additional application of an AMPAR antagonist, NBQX (from 895±260 to 367±84 pA, *n*=7, *P*=0.016, Fig. 6e, trace 4). Thus, the large baseline inward current in KO PCs is generated by AMPARs exposed to excessive external glutamate. A previous study showed that 200 μM DL-TBOA augmented AMPAR-mediated inward current of PCs in WT mice^19^, while KO PCs were sensitive enough to react to 50 μM DL-TBOA. These results further support the compromised glutamate-buffering/clearance in KO mice, which may be due to the delocalization of GLAST from PF–PC synapse active zones (Fig. 5e), and/or their dissociation from the perisynaptic clusters of septins and CDC42EP4 (Fig. 5a).

In addition, the content of glutamate, glutamine and related amino acids and monoamines in cerebellar tissues from WT and KO mice was comparable, as assessed using high-performance liquid chromatography (Table 2). Thus, the total amount of the glutamate/glutamine shuttle components, and other major neurotransmitters and metabolites, are unaffected by the loss of CDC42EP4.

### Impaired motor coordination/learning in *Cdc42ep4* ^*−/−*^ mice

**Figure 7:**
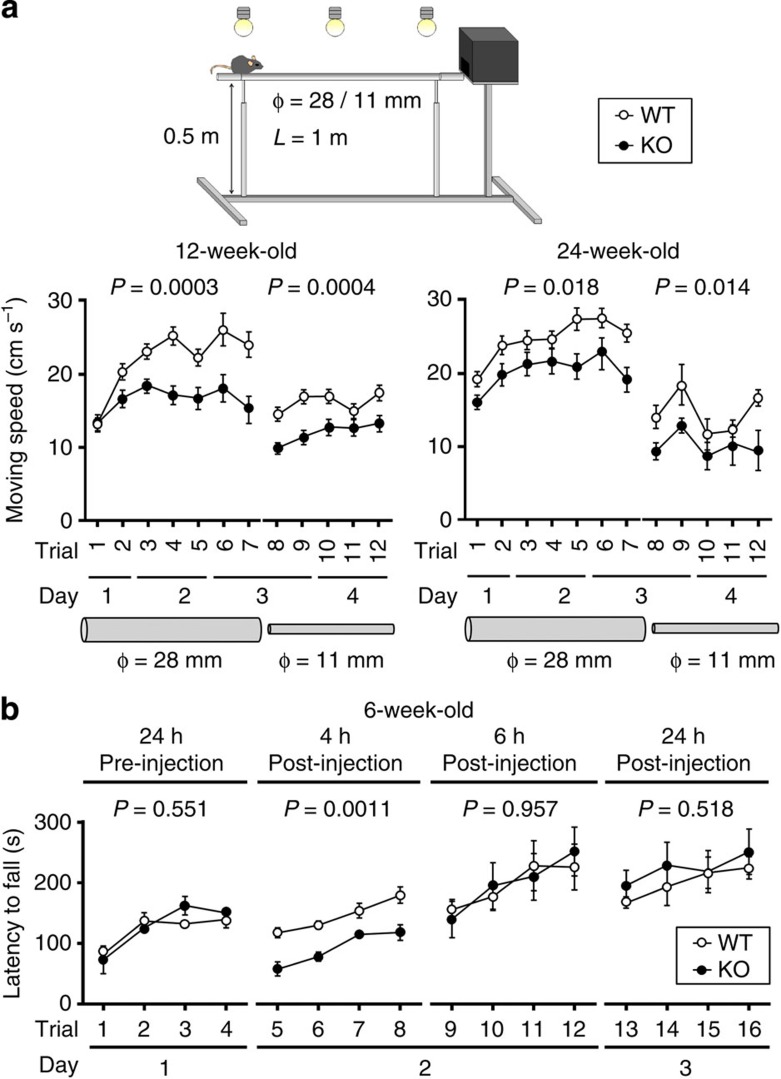
Motor coordination and motor learning defects in *Cdc42ep4*^−/−^ mice. (**a**; Top) Set-up for the balance beam test. The height (0.5 m above the floor) and illumination (100 lux) motivate mice to escape by traversing along a horizontal rod (1 m × 28 or 11 mm) into a dark box. (Bottom) The learning curves of a cohort of WT and KO mice measured at 3 and 6 months of age. The moving speeds are plotted for seven trials with a 28-mm rod and subsequent five trials with an 11-mm rod over 4 days. The motor coordination defects in KO mice remained uncompensated up to 6 months of age (*n*=13/13 and 12/12, *P*=0.0003, 0.0004, 0.018, 0.014 by two-way repeated measures ANOVA). (**b**) The learning curves of 6-week-old WT and KO littermate mice assessed by the rota-rod test before and after direct cerebellar cortical injection of CTZ plus DL-TBOA (100 μM each per 10 μl). The local inhibition of EAATs with the subthreshold dose of DL-TBOA elicited a significant motor coordination defects transiently (around 4 h post-injection) and only in KO mice (*n*=5/4, *P*=0.551, 0.0011, 0.957, 0.518 by two-way repeated measures ANOVA). The aberrant hypersensitivity of KO mice indicates their glutamate clearance deficit that is adaptively compensated.

As a means of unbiased screening for neural dysfunctions, a cohort of WT and KO littermates were subjected to a systematic battery of behavioural tests. In 14 behavioural test paradigms including the rotating rod (rota-rod) test, WT and KO mice performed almost comparably (Table 3 and Supplementary Figs 1–12). Given the robust compensatory potential of the motor control circuitry, and the molecular and electrophysiological endophenotype of KO mice, we assessed their motor coordination and motor learning with the balance beam test, which is superior to the rota-rod test in detection sensitivity^24^. While the moving speed of 12-week-old KO mice on the initial trial (the intercept) was normal, the slopes of their learning curves were significantly flatter than those of WT mice (*n*=13/13, *P*=0.0003 and 0.0004, respectively, for the beam diameters of 28 and 11 mm; Fig. 7a). In a follow-up of the same cohort at 24 weeks of age, the learning curve of KO mice was consistently lower than that of WT mice (*n*=12/12, *P*=0.018 and 0.014; Fig. 7a).

To test whether the persistent underperformance is caused by the insufficient glutamate-buffering/clearance capacity in Bergmann glia, and not by the loss of extracerebellar astrocytic CDC42EP4 (Fig. 2e), we attempted the partial inhibition of EAATs with DL-TBOA. Direct subpial injection of 100 μM DL-TBOA with CTZ above lobule VI of the cerebellar cortex did not affect the motor performance of WT mice, whereas the pharmacological decompensation elicited significant and transient motor incoordination in KO mice, as was obvious now in the rota-rod test (*n*=5/4, *P*=0.0011, Fig. 7b). The remarkable hypersensitivity to the subthreshold DL-TBOA recapitulated *in vivo* has corroborated the insufficient glutamate-buffering/clearance capacity in KO mice.

## Discussion

Previous studies indicated that CDC42EPs bind to small GTPases (CDC42, RhoQ/Tc10) and septins in a mutually exclusive manner^13^,^14^, and that interaction of CDC42EP1/BORG5 with CDC42 and atypical protein kinase C is required for cell motility in early mouse embryo^25^. A recent study revealed defective angiogenesis in *Cdc42ep1*^−/−^ mice, which is due partly to septin/actomyosin dysregulation that affects migration of vascular endothelial cells^26^. In contrast, CDC42EP4 in Bergmann glia is mainly in complex with septins and GLAST, and is dispensable for developmental cell migration and post-developmental morphological integrity. The distinct binding partners and physiological roles of CDC42EPs may reflect their distinct primary structure and/or expression pattern. Since *Cdc42ep1* mRNA is also expressed in Bergmann glia (Allen Mouse Brain Atlas #68342384), *Cdc42ep1*^−/−^ mice and *Cdc42ep1*^−/−^; *Cdc42ep4*^−/−^ double-mutant mice would exhibit intriguing cerebellar phenotypes.

Genetic loss of a vital septin subunit (that is, SEPT7, SEPT9 or SEPT11) causes embryonic lethality^27^,^28^,^29^, whereas obvious brain anomaly or motor incoordination has never been reported in mice that lack one or two other septin subunits (that is, SEPT3, SEPT4, SEPT5, SEPT6, SEPT3 and SEPT5, or SEPT4 and SEPT6), despite their abundance in the cerebellum and other brain regions^30^,^31^,^32^,^33^,^34^. In contrast, *GLAST*^−/−^ mice exhibit protracted glutamate transient, multiple CF–PC innervations and failure in the rota-rod test^6^,^21^.

The present study demonstrated that *Cdc42ep4*^−/−^ mice develop CF–PC innervations with largely normal wiring pattern and electrophysiological properties (Figs 3a and 6a), and pass the rotating rod (rota-rod) test (Table 3). However, pharmacological decompensation with inhibitors of AMPAR, AMPAR desensitization or EAATs consistently indicated their insufficient glutamate-buffering/clearance capacity in electrophysiological analyses (Fig. 6c–e), and caused failure in the rota-rod test (Fig. 7b). Thus, the severity of cerebellar phenotypes is ranked *GLAST*^−/−^>*Cdc42ep4*^−/−^>septin-null mice, which would justify an interpretation of the phenotype of *Cdc42ep4*^−/−^ mice as a mild insufficiency of GLAST function, rather than as a partial loss of septin function. Since *Sept4*^−/−^ mice and Bergmann glia-selective *Sept7*^−/−^ mice exhibit subnormal motor coordination only in infancy (Kinoshita, Ageta-Ishihara *et al*., unpublished), the pharmacological decompensation technique may help differentiate these and other septin mutant mice from WT littermates.

Glutamate released from PF boutons/terminals not only depolarizes PCs via AMPARs; however, the spillover fraction evokes Ca^2+^ responses in the perisynaptic compartments (‘microdomains') of lamellar Bergmann glial processes mainly via mGluR1 (refs 35, 36). A subdomain in such a compartment tightly enwraps a PF–PC synapse, where GLAST outnumbers other EAATs^4^,^37^. While EAAT4 is localized to PC spines for slow glutamate clearance^20^, GLAST is concentrated along perisynaptic processes of Bergmann glia and contributes to the fast and efficient limitation/termination of glutamate neurotransmission and spillover^1^,^5^,^20^. However, the molecular basis to target and concentrate GLAST to the proximity of synaptic gap remains unclear.

Physical and functional interactions of the cytoplasmic tail of GLAST with submembranous septins have been consistently demonstrated *in vitro*, in tissue culture cell cortex and Bergmann glial processes^11^,^12^. The interaction with submembranous septins has been hypothesized to provide membrane-bound GLAST trimers with scaffolds, limit their lateral diffusion and/or augment their transport activity, which collectively contribute to perisynaptic concentration of glutamate clearance activity. However, the hypothesis has never been tested *in vivo* partly because of the aforementioned redundancy problem in septin reverse genetics.

This study demonstrated that CDC42EP4 clusters beneath Bergmann glial membrane subdomains enwrapping dendritic spines where septins and GLAST abound^4^,^11^,^16^. In the absence of CDC42EP4, GLAST is dissociated from septins (Fig. 5a) and delocalized away from synapses (Fig. 5e). These findings support the above hypothesis of septins as scaffold/barrier for GLAST, and may further implicate subtle alterations in tripartite synapse geometry, for example, retraction of glial components from synaptic clefts, with or without secondary remodelling of neuronal components.

The distribution of GLAST visualized with IF and immunoelectron microscopy appears more diffuse along Bergmann glial membranes than that of CDC42EP4 (Figs 1f, 4e and 5c), which corresponds to the IP/IB data that only a subset of GLAST is pulled down with CDC42EP4 and/or septins. These data suggest other membrane-proximal proteins that contribute to perisynaptic localization of GLAST. One such candidate is NHERF1 (Na^+^/H^+^-exchange regulatory factor-1) whose PDZ domain interacts with the cytoplasmic tail of GLAST^12^,^38^. So far, however, neither GLAST-related phenotype for *Nherf1*^−/−^ mice^39^ nor expression and localization NHERF1 in Bergmann glia has been reported. Thus, the unique phenotype of *Cdc42ep4*^−/−^ mice provides the first *in vivo* evidence for the significance of perisynaptic concentration of GLAST by submembranous scaffold and/or diffusion barrier.

GLAST abounds near PF–PC synapses and the buffering/clearance capacity far surpasses the level of glutamate release, which confers remarkable tolerance to EAAT inhibitors. Our electrophysiological analysis with pharmacological interventions revealed insufficient glutamate-buffering/clearance capacity in *Cdc42ep4*^−/−^ PF–PC synapses. The decay time constant of EPSC, which reflects the retention of extracellular glutamate, was mildly protracted on inhibition of AMPAR desensitization with CTZ (Fig. 6c). Additional inhibition of EAATs with subthreshold DL-TBOA significantly augmented AMPAR-mediated inward current only in the *Cdc42ep4*^−/−^ cerebellum (Fig. 6d,e). Further, a low-affinity glutamate analogue γDGG was less effective in the inhibition of EPSC (Fig. 6d). These data concordantly indicate higher extracellular glutamate level in the mutant. Given the properties of EAATs and their distribution around glutamatergic synapses to PCs^5^,^20^,^37^, and unaltered expression of the major AMPAR subunits in the *Cdc42ep4*^−/−^ cerebellum (Fig. 3d), the phenotype is attributed principally, if not all, to the insufficiency of GLAST function. As the total amounts of glutamate and GLAST are unaltered in the *Cdc42ep4*^−/−^ cerebellum, the delocalization of GLAST from perisynaptic glial processes (Fig. 5e), with or without altered transporter activity due to the dissociation from CDC42EP/septins, appears to be the major cause of the glutamate intolerance.

Overall, the unique phenotype of *Cdc42ep4*^−/−^ mice provided the first *in vivo* evidence for submembranous molecular network that is required for the perisynaptic concentration and efficiency of GLAST. This study illuminated the spatial regulation of GLAST by the CDC42EP4/septin-based scaffold beneath perisynaptic glial membranes, which ensures optimal buffering/clearance of extracellular glutamate from around synapses. Since CDC42EPs and septins, as with GLAST, are implicated in human neurological and psychiatric disorders including schizophrenia by unbiased proteomic and genome-wide association studies^8^,^9^,^40^,^41^,^42^,^43^, whether relevant mechanisms underlie the pathophysiology in other brain regions is to be tested in future studies.

## Methods

### Animal experiments

Animal experiments had been approved by the institutional review committees and conducted in accordance with the regulations for the care and use of animals at Nagoya University, Niigata University, Hokkaido University, Hiroshima University, Fujita Health University and Gunma University. We consistently compared male littermates raised in the same cages unless otherwise noted.

### Antibodies

We raised rabbit polyclonal antibodies against seven antigens and consistently used one (#2) against a recombinant polypeptide Arg^58^–Met^100^ from CDC42EP4 that gave the best signal/noise ratio in IB and IF analyses. The specificity was warranted by the absence of the signals in *Cdc42ep4*^−/−^ tissues (Fig. 2d,e). We used antibodies for septins, SEPT2 (1:2,000), SEPT4 (1:3,000) and SEPT7 (1:4,000) as previously described^33^,^44^, GLAST^45^, 3-phosphoglycerate dehydrogenase (Phgdh)^46^, calbindin^47^, carbonic anhydrase 8 (Car8)^48^, VGluT1, 2 (ref. 49) and commercial antibodies for GluR1, 2 (Alomone Labs, AGC-004, 1:200, AGC-005, 1:150), GLAST (Frontier Institute, Rb-Af660, 1:2,000), PSD-95 (Cell Signaling, 3450, 1:1,000), CDC42 (Santa Cruz, L0809, 1:100), β-actin (Sigma, A5441, 1:5,000) and α-Tubulin (Sigma, T9026, 1:10,000). For secondary antibodies, we used Alexa 488-, Cy3- or horseradish peroxidase-conjugated IgGs from rabbit, goat or guinea pig (Jackson ImmunoResearch, 706-545-148, 711-165-152, 111-035-003) diluted at 1:200–1,000. Histidine-tagged recombinant CDC42 (Cytoskeleton) was used to quantify endogenous CDC42.

### Biochemical fractionation and IB analysis

Each tissue was dissected, weighed and homogenized by sonication in 3 ml g^−1^ of buffer A (10 mM Tris-HCl at pH 7.6, 0.15 M NaCl, 1% Triton X-100, protease inhibitors). The supernatant after centrifugation at 20,400*g* at 4 °C for 0.5 h was labelled as soluble fraction. The pellet was dissolved with sonication in 1 ml g^−1^ buffer B (3% SDS, 5% 2-mercaptoethanol), which was termed pellet fraction. The samples were resolved by 10%PAGE (SDS-PAGE), transferred to reinforced nitrocellulose or PVDF membranes and subjected to IB analysis using a blocking buffer containing 5% skim milk in TBST (0.1 M Tris-HCl at pH 7.4, 0.15 M NaCl, 0.05% Tween 20). Chemiluminescence detection and densitometry were performed with horseradish peroxidase-conjugated secondary antibodies, ECL-Plus reagent (PerkinElmer), TrueBlot (Rockland) and an image analyser LAS-3000mini with MultiGauge software (Fuji). Uncropped version of each image is shown in Supplementary Fig. 15.

### Immunoprecipitation

For IP/IB experiments, whole cerebella were lysed with buffer A' (0.2 M Tris-HCl at pH 7.35, 0.3 M NaCl, 0.2% SDS, 0.2% Triton X-100, 0.2% sodium deoxycholate, 20 mM 2-mercaptoethanol and protease inhibitors), centrifuged and the supernatant was mixed with protein A Sepharose CL-4B (GE Healthcare) or Affi-Prep Protein A Support (Bio-Rad) that had been preincubated with anti-CDC42EP4, anti-SEPT4 or nonimmune rabbit IgG (as a negative control). After washing the beads with the same buffer, bound proteins were lysed for IB. For IP/MS experiments, we used buffer C (20 mM Tris-HCl at pH 8.0, 0.15 M NaCl, 1 mM EDTA, 1% NP-40 and protease inhibitors) with more stringent wash condition^50^.

### Mass spectrometry and peptide mass fingerprinting

After IP, bead-bound proteins were eluted with solution (7 M guanidine, 50 mM Tris-HCl at pH 8.0), reduced with 5 mM dithiothreitol for 0.5 h, alkylated with 10 mM iodoacetamide for 1 h in the dark, demineralized and concentrated by methanol/chloroform precipitation and digested (0.5 μg trypsin in 1.2 M urea and 50 mM Tris-HCl at pH 8.5).

Mass spectrometric analysis (LC-MS/MS) was carried out as previously described^50^. Briefly, we used an LTQ Orbitrap XL mass spectrometer (Thermo Scientific) connected to an HTC-PAL autosampler and a Paradigm MS4 HPLC with a C18 reversed-phase column and ADVANCE Plug and Play Nano Source (Michrom Bioresources). The reversed-phase chromatography condition was a linear gradient (0 min, 5% B; 70 min, 100% B) of solvent A (2% CH_3_CN with 0.1% trifluoroacetic acid (TFA)) and solvent B (98% CH_3_CN with 0.1% TFA) at a flow rate of 500 nl min^−1^. The mass spectrometer was operated in the data-dependent MS2 mode. Peak lists were generated and calibrated by using the Mascot software (Matrix Science), and validation of the MS/MS-based peptide and protein identifications was made with the Scaffold programme (ver. 3, Proteome Software). The peptide probability by Mascot was used for the Scaffold Local FDR algorithm. Peptide identification was approved by the presence of at least two peptides that exceeded the probability of 95%. Proteins that contained similar peptides and could not be differentiated by MS/MS data alone were grouped to satisfy the principle of parsimony.

### Measurement of neurotransmitters and metabolites

We used previously described methods for the extraction and measurement of amino acids, amines and metabolites^51^,^52^,^53^,^54^. Briefly, cerebellar hemispheres dissected on ice were frozen in liquid N_2_ and stored at −80 °C until extraction. For measurement of amino acids, each sample was weighed, homogenized in 1.5 ml CH_3_OH on ice, centrifuged at 3,000*g* for 6 min at 4 °C and 20 μl of the supernatant was dried by evaporation at 40 °C. The residue was dissolved with 20 μl water, 20 μl 0.1 M borate buffer at pH 8.0 and 60 μl 50 mM 4-fluoro-7-nitro-2,1,3-benzoxadiazole (NBD-F; Tokyo Kasei Kogyo) in CH_3_CN, reacted at 60 °C for 2 min and stopped by adding 100 μl 0.1% TFA and 10% CH_3_CN in water. Total D-/L-serine levels were measured using a column-switching HPLC system (SCL-10A VP, Shimadzu)^51^. Glutamate, glutamine, glycine and GABA were measured using an HPLC system (LC-20AT, Shimadzu)^52^. Fluorescence emission at 530 nm was measured with an excitation at 470 nm. For monoamines and their metabolites, tissue samples were homogenized in 0.2 M HClO_4_ containing 0.1 mM EDTA and 100 μg l^−1^ isoproterenol (internal standard), and centrifuged at 20,000*g* for 15 min at 4 °C. Supernatants were filtered through 0.45-μm pore membranes (Millex-LH, Millipore). We used a reversed-phase column (SC-5ODS) and an electrochemical detector (ECD-300) in an HPLC system (EP-300, DG-300 and EPC-300, all from Eicom) with the mobile phase containing 0.1 M acetate–citric acid buffer at pH 3.5, 16% CH_3_OH, 5 mg l^−1^ EDTA and 0.19 g l^−1^ sodium octyl sulfate.

### Tissue preparation for histochemistry

Under deep pentobarbital anaesthesia (0.1 mg per g of body weight), mice were fixed transcardially with 4% paraformaldehyde in 0.1 M phosphate buffer (PB) at pH 7.2 for IF and immunoelectron microscopy, or with 4% paraformaldehyde/0.1% glutaraldehyde in PB for TEM. After excision from the skull, brains for immunohistochemistry were immersed in the same fixative for 2 h. In IF for CDC42EP4, fixed brains were sliced at 50 μm with a microslicer (VT1000S, Leica) and subjected to free-floating incubation, while 4-μm-thick paraffin sections with a sliding microtome (SM1000R, Leica) were used for Car8, GLAST, VGluT1 and VGluT2. Brains for FISH were removed under deep pentobarbital anaesthesia and immediately frozen in powdered dry ice, cut at 20 μm with the cryostat and mounted on silane-coated glass slides.

### FISH

Mouse cDNA fragments for *Cdc42ep4* (nucleotide 331–1,118, GenBank BC003857), calbindin (37–1,071, BC016421), GLAST (1,620–2,576, BC066154) and Phgdh (1–1,799, BC110673) were subcloned into the pBluescript II plasmid vector. Digoxigenin (DIG)- or fluorescein-labelled cRNA probes were transcribed *in vitro*^55^.

Sections were processed as follows: acetylation with 0.25% acetic anhydride in 0.1 M triethanolamine-HCl at pH 8.0 for 10 min and prehybridization for 1 h in buffer A (50% formamide, 50 mM Tris-HCl at pH 7.5, 0.6 M NaCl, 0.02% Ficoll, 0.02% polyvinylpyrrolidone, 0.02% bovine serum albumin, 200 mg l^−1^ tRNA, 1 mM EDTA and 10% dextran sulfate). Hybridization with cRNA probes was performed in buffer A at 63.5 °C for 12 h, and then sections were washed at 61 °C with 5 × standard sodium citrate (SSC) for 0.5 h, 4 × SSC containing 50% formamide for 0.7 h, 2 × SSC containing 50% formamide for 0.7 h and 0.1 × SSC for 0.5 h. Sections were incubated successively in NTE buffer (0.5 M NaCl, 0.01 M Tris-HCl at pH 7.5, 5 mM EDTA), 20 mM iodoacetamide in NTE buffer and TNT buffer (0.1 M Tris-HCl at pH 7.5, 0.15 M NaCl), each for 0.3 h at room temperature.

For double-labelling FISH, sections were reacted with peroxidase-conjugated anti-fluorescein antibody (Invitrogen, A21253, 1:1,500) and incubated with the FITC-TSA plus amplification kit (PerkinElmer). After inactivation of residual peroxidase activities with 3% H_2_O_2_, the sections were incubated successively with DIG-labelled cRNA probe, the peroxidase-conjugated anti-DIG IgG (Roche, 11 207 733 910, 1:1,000) and the Cy3-TSA Plus reagents (PerkinElmer), and then counterstained with TOTO-3 (Invitrogen, T3604, final 0.2 μM) in PBS.

### Immunohistochemistry

For immunohistochemistry, 4-μm-thick paraffin sections were incubated with 10% normal donkey serum for 20 min, a mixture of primary antibodies (1 mg l^−1^) overnight and a mixture of Alexa 488- or Cy3-labelled species-specific secondary antibodies for 2 h at a dilution of 1:200. Fluorescence imaging was conducted using scanning laser confocal microscopes IX81/FV1000 (Olympus) with a × 40 objective lens (numerical aperture (NA) 1.0) and LSM-780 (Zeiss) with × 40 (NA 1.1) and × 63 (NA 1.4) objective lenses, and wide-field microscopes (BZ-9000, Keyence or BX-60, Olympus) with × 40 and × 20 objective lenses (NA 1.3, Nikon or NA 0.7, Olympus).

### Electron microscopy

Conventional electron microscopy, pre-embedding silver-enhanced immunoelectron microscopy and postembedding immunogold electron microscopy were conducted as described previously^56^,^57^. Briefly, pre-embedding immunoelectron microscopy was conducted by incubation in blocking solution for goat gold conjugates (Aurion, Electron Microscopy Sciences) for 0.5 h, and then with CDC42EP4 antibody (1 mg l^−1^) diluted with 1% BSA and 0.004% saponin in PBS overnight. Anti-rabbit IgG linked to 1.4 nm gold particles (1:100, Nanogold labelling reagents, Nanoprobes, #2003) were incubated for 2 h, and gold particles were intensified using a silver enhancement kit (R-Gent SE-EM, Aurion). In post-embedding immunogold, 80-nm-thick sections were cut with an ultramicrotome (Ultracut, Leica), incubated overnight with rabbit GLAST antibody (20 mg l^−1^) and then with colloidal gold (10 nm)-conjugated anti-rabbit or anti-guinea pig IgG (British Biocell, EM.GAR10, 1:100) for 2 h. The samples were mounted on grids and stained with 2% uranyl acetate for 20 min, and observed with a TEM (H-7100, Hitachi).

### Electrophysiology

We prepared 250-μm-thick cerebellar slices as described previously^58^,^59^. In brief, 5–7-week-old mice deeply anaesthetized with CO_2_ were decapitated. The cerebella were quickly cut parasagittally with a microslicer (VT1200S, Leica) in a chilled cutting solution ((in mM) 120 choline-Cl, 3 KCl, 1.25 NaHPO_4_, 28 NaHCO_3_, 8 MgCl_2_ and 25 glucose), incubated in normal ACSF (artificial cerebrospinal fluid; 125 NaCl, 2.5 KCl, 2 CaCl_2_, 1 MgSO_4_, 1.25 NaH_2_PO_4_, 26 NaHCO_3_ and 20 glucose, bubbled with 95% O_2_ and 5% CO_2_) at 36 °C for 0.5 h and then kept at room temperature. All recordings were performed at 32–34 °C. Whole-cell recordings were made from visually identified PCs under an upright microscope (BX51WI, Olympus). Pipette solution contained (55 CsCl, 10 Cs D-gluconate, 20 TEA-Cl, 20 BAPTA, 4 MgCl_2_, 4 ATP, 0.4 GTP, 5 QX314-Cl and 30 HEPES at pH 7.3, adjusted with CsOH). To block inhibitory synaptic transmission, 10 μM bicuculline was consistently supplemented to the normal ACSF. All the drugs were obtained from Tocris Bioscience. Stimulation pipettes (∼5 μm tip diameter) filled with the normal ACSF were used to apply square pulses for focal stimulation (duration, 0.1 ms; amplitude, 0–90 V). PFs were stimulated in the molecular layer. CFs were stimulated in the granular layer around the PC soma under investigation. The stimulation was given at 0.2 Hz (Fig. 6a,b) or 0.05 Hz (Fig. 6c–e). The number of CFs innervating the recorded PC was estimated by the number of discrete CF-EPSC steps in response to a gradual rise in the stimulus intensity. The stimulation pipette was systematically moved around each PC soma. Ionic currents were recorded with a patch clamp amplifier (EPC10, HEKA Elektronik). The pipette access resistance was compensated by 70–80%. Online data acquisition and offline data analysis were conducted with software, PATCHMASTER and FITMASTER (HEKA).

### Generation of *Cdc42ep4*-floxed and null mice

The targeting strategy is illustrated in Fig. 2a. We constructed the targeting vector using the MultiSite Gateway Three-Fragment Vector Construction kit (Invitrogen) and BAC Subcloning Kit (Gene Bridges) with some modifications. Briefly, a DNA fragment carrying the loxP sequence and *pgk*-promoter-driven neomycin-resistance (Neo) cassette flanked by two Flp recognition target sites was inserted in reverse orientation directly downstream of the sole coding exon of *Cdc42ep4* isolated from a C57BL/6 mouse genomic BAC clone RP23-366J17. The other loxP site was inserted into the intron directly upstream of the coding exon. The resulting targeting vector, which consisted of 5′- and 3′-homology arms, the floxed coding region with the Neo cassette and diphtheria toxin gene (DT) for negative selection, was linearized and electroporated into the C57BL/6N mouse embryonic stem (ES) cell line, RENKA^60^. Genomic DNA from neomycin/G418-resistant ES cell clones were analysed with Southern blot analysis, and positive clones with proper homologous recombination were microinjected to host embryos as described previously^61^. The null allele (*Cdc42ep4*^−^) was created by crossing heterozygous floxed (*Cdc42ep4*^fl/+^) mice with CAG-Cre transgenic mice^62^. For Southern blot analysis, genomic DNA was digested with Sac I, EcoR V and Hinc II, and hybridized with 5′, 3′ and neo probes, respectively. The predicted fragment sizes for the WT and floxed alleles for each probe, 13/11.3, 16.4/12.7 and N.A./16.1 (kb), respectively, were confirmed. We thereafter conducted genotyping using PCR with the following primers: (a) 5′-TGCTTCAGTACCTTCGGAC-3′, (b) 5′-TTCGAGTTCACAGAGCTGGA-3′ and (c) 5′-TCATAGAGAAGGTGGCAGC-3′ to distinguish the WT, floxed and null alleles by 490-bp (with primers a+b), 580-bp (a+b) and 480-bp (a+c) products, respectively. After confirming germline transmission and Mendelian inheritance of the *Cdc42ep4*^fl^ and *Cdc42ep4*^−^ alleles, the lines have been deposited to RIKEN Bioresource Center (RBRC04894 and RBRC09539, respectively).

### Balance beam and rota-rod tests

All systematic behavioural tests were conducted on male littermates (10–38 weeks old) as described previously^33^,^63^. In the balance beam test, the motor coordination was quantitatively assessed by the performance on the first trial, the moving speed and the numbers of pauses and slips along a 1-m-long rod (diameter, 28 mm). After six trials on Days 1–3, five trials with a thinner rod (diameter, 11 mm) were conducted on Days 3–4. The increment in the moving speed and decrements in the numbers of pauses and slips were quantified as indices of motor learning. In the rota-rod test, we measured the latency to fall from a rotating rod (diameter, 30 mm) that was accelerated from 4 to 40 r.p.m. over 5 min per trial. For pharmacological experiment (see below), each mouse was subjected to four sessions (four trials per session) at 24 h before injection, and 4, 6 and 24 h after injection.

### Direct cerebellar cortical injection

We applied a method established for viral delivery^64^ with minor modifications. Briefly, 6-week-old mice (WT, 4F+1M; KO, 3F+1M) anaesthetized with ketamine (100 mg kg^−1^) and xylazine (16 mg kg^−1^) were mounted in a stereotactic frame, and the occipital bone was drilled at 5 mm caudal to the bregma. A blunt-ended Hamilton syringe tip (33 G) attached to a micropump and a controller (UltramicroPump II, Micro4; World Precision Instrument) was placed beneath the pia mater above lobule VI. The mixture of CTZ and _DL_-*threo*-β-benzyloxyaspartic acid (DL-TBOA; 100 μM each in 10 μl) was injected at a rate of 333 nl min^−1^. After suturing the scalp, and recovery from anaesthesia on a warm pad, the mice were returned to home cages.

### Systematic behavioural analysis

We applied our standard protocols^63^,^65^,^66^ to a cohort of male littermates (*n*=13, 13) reared in the same cages: behavioural testing was conducted between 9 a.m. and 6 p.m. except for the continuous home cage monitoring. Each apparatus was cleaned with sodium hypochlorite solution to minimize odour after use. We conducted tests in the following order: general health and neurological screening (including body weight and temperature measurements, grip strength test, righting test, whisker touch test and ear twitch reflexes, wire hang test), light/dark transition test, open field test, elevated plus maze test, one-chamber social interaction test, rota-rod test, three-chamber sociability and preference for social novelty test, prepulse inhibition test of acoustic startle response, Porsolt forced swim test, Barnes maze test, tail suspension test and long-term monitoring of locomotion and social interaction in home cage. Intervals between tests were >24 h.

### Neuromuscular strength tests

Neuromuscular strength was assessed with the forelimb grip strength test and wire hang test. Forelimb grip strength was measured by pulling a mouse in the tail while its forepaws hung on to a wire grid attached to a spring balance. The tensile force (*N*) when the mouse released the grid was measured three times, and the greatest value was analysed. In the wire hang test, a wire mesh with a mouse on top was slowly inverted and the latency to fall was measured.

### Light/dark transition test

The apparatus had a pair of differentially illuminated (390 versus 2 lux) chambers (21 × 41 × 25 cm) connected with a door in the middle. Each mouse was released in the dark chamber, and image data were acquired from the top with a CCD (charge-coupled device) camera for 10 min. The latency until the first entry into the light chamber, the time spent in each chamber, the number of transitions and the total distance travelled were automatically measured using ImageLD software (see Image analysis).

### Open field test

Voluntary locomotor activity was measured in an open field test. Each mouse was placed in the centre of the open field apparatus (40 × 40 × 30 cm; Accuscan Instruments) illuminated at 100 lux. The following indices were monitored for 120 min: total distance travelled, time spent in the centre area of 20 × 20 cm, number of rearing and beam-breaks were automatically measured by counting interruptions of infrared beams.

### Elevated plus maze test

The apparatus had two open arms (25 × 5 cm, with 3-mm-high plastic ledges) and two closed arms (25 × 5 cm, with 15-cm-high transparent walls) interconnected via a central crossing (5 × 5 cm), which was set at 55 cm height and illuminated at 100 lux. The numbers of entries into, and the time spent in the open and enclosed arms, were recorded for 10 min. Image data were acquired from the top with a CCD camera, and the number of entries into and the time spent in the open/closed arms, and total distance travelled, were measured automatically using the ImageEP software (see Image analysis).

### Acoustic startle response and prepulse inhibition test

A mouse restrained in a cylinder was placed in the chamber of a startle reflex measurement system (O'Hara & Co.) with 70-dB background white noise. After 10 min, the mouse's startle response to a startle stimulus (110 or 120 dB white noise for 40 ms) was measured by a motion sensor for 140 ms. A test session was a random sequence of four trials each with a prepulse stimulus (74 or 78 dB white noise for 20 ms that preceded the startle stimulus by 100 ms) and two without. Six blocks of six trials were presented in a pseudorandom order with the average intertrial interval of 15 s.

### Porsolt forced swim test

Each mouse was released in 7.5-cm-deep water at 23 °C in an acrylic cylinder (10 cm in diameter), and the duration of the motion for evacuation was measured up to 10 min automatically using the ImageTS/PS software (see Image analysis).

### Social interaction and voluntary activity in the home cage

The position of each mouse housed alone in a cage was monitored from the top continuously for a week. The distance travelled along the diurnal cycle was measured automatically using the ImageHA software (see Image analysis). Two mice of the same genotype that had been separately reared were housed together in a home cage and their two-dimensional (2D) images from the top were captured at 1 fps for a week. Their physical contact and separation were represented, respectively, as one and two particles, and their locomotor activity was quantified by the differentials of pixels between successive frames by using the ImageHA software (see Image analysis).

### One-chamber social interaction test

The positions of two mice placed in a novel chamber (40 × 40 × 30 cm) were monitored from the top at three frame s^−1^. Their horizontal distance travelled and the number of contacts were measured automatically using the ImageSI software (see Image analysis).

### Three-chamber test for sociability and social novelty

The apparatus for Crawley's test had three chambers (20 × 40 × 22 cm) separated by two transparent partitions each with an opening (5 × 3 cm) and a lid with an infrared CCD camera. A male mouse (9-week old, C57BL/6J, termed Stranger 1) that had no prior contact with the subject mice was enclosed in a cylinder cage (9 cm in diameter, set in the left chamber) that allowed nose contacts. Each subject mouse was released in the middle chamber and allowed to explore for 10 min, while the time spent in each chamber and within 5 cm from each cage was measured automatically using the ImageCSI software (see Image analysis). Subsequently, another unfamiliar mouse (Stranger 2) was placed in another cylinder cage (in the right chamber) and monitored likewise for another 10 min.

### Rota-rod test

For the initial screening, each mouse was subjected to nine trials over 3 days. See Balance beam and rota-rod tests for details.

### Tail suspension test

The movement of each mouse suspended by the tail at a height of 30 cm was recorded for 10 min and analysed by using the ImageTS/PS software (see Image analysis).

### Barnes maze test

The apparatus consisted of a blight (800 lux), white circular platform (diameter, 1 m) with 12 holes along the perimeter, 0.75 m above the floor, and a dark escape box (containing cage bedding) set under one of the holes (‘target'). Following habituation sessions on the day before, each mouse was consistently trained to learn a randomly assigned target position (1–3 trials per day × 6 days). The platform was turned 90° per day to minimize the influence from local cues. The distance travelled, time spent and number of errors from the centre to the target hole were measured from time-lapse images with a video-tracking software, ImageBM. One day (24 h) after the last training session, the first probe test was carried out without escape box to exclude local cue-dependent navigation, when mice were allowed to explore for 3 min and the time spent around each hole was measured. The probe test was followed by a training session with the target. To assess long-term spatial memory retention, the mice were again subjected to the probe test 1 month later.

### Image analysis

The application programmes for behavioural data acquisition and analysis (ImageLD, EP, SI, CSI, TS/PS, BM, HA) were created on the ImageJ (http://rsb.info.nih.gov/ij/). ImageLD and EP are freely available from http://www.mouse-phenotype.org/software.html.

### Statistical analysis

Quantitative data are represented as mean±s.e.m. Prism 4.0 (GraphPad Software), Stat View (SAS Institute) or Sigma Plot (SYSTAT), Excel (Microsoft) were used for statistical analyses. Behavioural data were analysed by analysis of variance (ANOVA; one-way and two-way repeated measures), unless otherwise noted. For the other data, *t*-test, Mann–Whitney *U*-test, Wilcoxon signed-rank test (two rightmost plots in Fig. 6e) and two-way (Fig. 6c) or two-way repeated measures ANOVA (Fig. 6e) followed by Tukey's *post hoc* comparisons were applied. For behavioural tests, either one-way ANOVA or two-way repeated measures ANOVA was applied for statistical analyses unless otherwise noted.

## Additional information

**How to cite this article:** Ageta-Ishihara, N. *et al*. A CDC42EP4/septin-based perisynaptic glial scaffold facilitates glutamate clearance. *Nat. Commun*. 6:10090 doi: 10.1038/ncomms10090 (2015).

## Supplementary Material

Supplementary Information Supplementary Figures 1-15 and Supplementary Table 1

## Figures and Tables

**Table 1:** Proteomic analysis for the binding partners of CDC42EP4 in the cerebellum.

Identified proteins (69)	Peptide number				Sequence coverage (%)			
	KO IgG	KO ep4	WT IgG	WT ep4	KO IgG	KO ep4	WT IgG	WT ep4
CDC42EP4				20				63
SEPT7			1	21			2.1	45
SEPT4			1	19			1.9	43
SEPT11				14				34
SEPT5				12				42
SEPT2				11				47
α-II spectrin			8	18			4.7	9.1
SEPT3				7				22
Myosin-10	2		1	10	1.3		0.81	6.7
SEPT8				6				24
SEPT10				5				16
SEPT6				5				26
β-II spectrin			1	4			0.8	2.8

List of proteins purified from WT cerebellar lysate with CDC42EP4-immunoaffinity column and identified LC-MS/MS analysis (rightmost columns). The numbers denote the count of peptides assigned to each protein (left) and the sequence coverage (right). The blank columns are zero. The specificity of the counts is corroborated by few false-positive counts in the controls (three left columns, that is, KO lysate captured with nonimmune IgG or anti-CDC42EP4 antibody, and WT lysate captured with nonimmune IgG). Another co-IP method used for the immunoblot analyses detected GLAST (peptide number=2, sequence coverage=6.45), but not CDC42. Co-IP, Co-immunoprecipitation; KO, knockout; WT, wild type.

**Table 2:** The bulk content of representative neurotransmitters and metabolites in the cerebella of *Cdc42ep4*^fl/fl^ and *Cdc42ep4*^−/−^ littermate mice.

Neurotransmitters and metabolites (per mg tissue)		
	WT	KO
Glu (nmol)	8.07±0.521	7.97±0.253
Gln (nmol)	5.54±0.459	5.25±0.128
Gly (nmol)	0.66±0.065	0.81±0.13
GABA (nmol)	1.43±0.102	1.48±0.133
L-Ser (nmol)	0.40±0.028	0.40±0.028
D-Ser (nmol)	0.003±0.0001	0.003±0.0000
5-HT (ng)	0.16±0.021	0.16±0.013
5-HIAA (ng)	0.09±0.01	0.11±0.007
NE (ng)	0.35±0.037	0.39±0.013
MHPG (ng)	0.12±0.062	0.07±0.0006
HVA (ng)	0.005±0.003	0.008±0.002
DA (ng)	0.004±0.001	0.005±0.003
DOPAC (ng)	0.006±0.002	0.008±0.004

The amount of glutamate, glutamine and other substances extracted from WT and KO cerebellar tissues (*n*=3, 3). There was no significant difference. KO, knockout; WT, wild type.

**Table 3:** Systematic behavioural test results of *Cdc42ep4*^fl/fl^ and *Cdc42ep4*^−/−^ littermate mice.

Tests	Mental/physical activities	Indices measured (inexhaustive)	Alteration from wild-type *Cdc42ep4*^−/−^	Related figures*
General health and	General health	Body weight	→	1
neurological screening		Rectal temperature	→	
		Grip strength	→	
		Hanging persistence	→	
Light/dark transition test	Exploratory activity	Distance travelled in the light chamber	→	2
	Light avoidance	Distance travelled in the dark chamber	→	
		Latency to the first entry to the light chamber	→	
		Time stayed in the light chamber	→	
		Number of transitions between chambers	→	
Open field test	Exploratory activity	Distance travelled	→	3
	Avoidance from open space	Centre time	→	
	Anxiety-like behaviour	Vertical activity	→	
		Stereotypical movements	→	
Elevated plus maze test	Exploratory activity	Distance travelled	→	4
	Height avoidance	Entries into open arms	→	
		Number of entries	→	
		Time stayed on open arms	→	
Acoustic startle response	Startle reflex to loudness	Amplitude of body motion	→	5a
Prepulse inhibition (PPI) test	Sensorimotor gating	Decrement of startle amplitude	→	5b
Porsolt forced swim test	Despair-like behaviour	Latency to immobility	→	6
Home cage monitoring	Diurnal cycle of locomotor activity	Activity level (distance travelled)	→	7
	Social behaviour	Mean number of particles	→	
Social interaction test (one-chamber, stranger pair)	Social behaviour, anxiety-like behaviour	Distance travelled	→	8
		Number of contacts	→	
		Total duration of active contacts	→	
		Mean contact duration	→	
		Total duration of contacts	→	
Social interaction test (three chambers, one to two caged strangers	Social behaviour, anxiety-like behaviour	Time spent with novel stranger	→	9
		Distance travelled	→	
Rota-rod test	Motor coordination/learning	Latency to fall	→ (↓ with CTZ+TBOA)	Fig. 7b, 10
Tail suspension test	Behavioural despair	Latency to immobility	→	11
Barnes maze test	Spatial memory	Time spent around target hole	→	12
Balance beam test	Motor coordination/learning	Moving speed	↓	Fig. 7a

Comparative table of the results of systematic behavioural tests with WT and KO littermate mice (C57BL/6, male, *n*=13-8/13-9 (the dropouts are due to failed data acquisition or spontaneous deaths)). Symbols (KO phenotype relative to WT): →, no significant difference; ↑/↓, statistically significant increase/decrease (*P*<0.05). *See Figs. 7a,b and Supplementary Figs 1–12 for details.
