# Supplementary material for: A CDC42EP4/septin-based perisynaptic glial scaffold facilitates glutamate clearance
Source: Nat Commun. 2015 Dec 10;6:10090. doi: 10.1038/ncomms10090 (PMC4682051; doi:10.1038/ncomms10090)
Supplement: Supplementary Information — Supplementary Figures 1-15 and Supplementary Table 1 [file ncomms10090-s1.pdf]

Kinetics of CF-EPSC

	Amplitude (pA)	10-90% rise time (ms)	Decay τ (ms)	
**Wild-type mice**	1294 ± 86	0.49 ± 0.03	8.13 ± 0.38	(n=16)
**Knockout mice**	1394 ± 105	0.52 ± 0.03	8.08 ± 0.31	(n=17)
	p=0.55	p=0.44	p=0.97	

Paired-pulse ratio of the PF-EPSC in response to paired stimuli at 50-ms interval

	Paired-pulse ratio	
**Wild-type mice**	1.73 ± 0.04	(n=22)
**Knockout mice**	1.73 ± 0.04	(n=19)
	p=0.824	

Supplementary Table 1. Comparison of the major kinetic parameters of evoked excitatory postsynaptic current (EPSC) of Purkinje cells by the stimulation of climbing fibers (CF), and the ratio of paired-pulse facilitation by the stimulation of parallel fibers (PF). These indices were comparable between young adult WT and KO mice. See Fig. 6 for details.

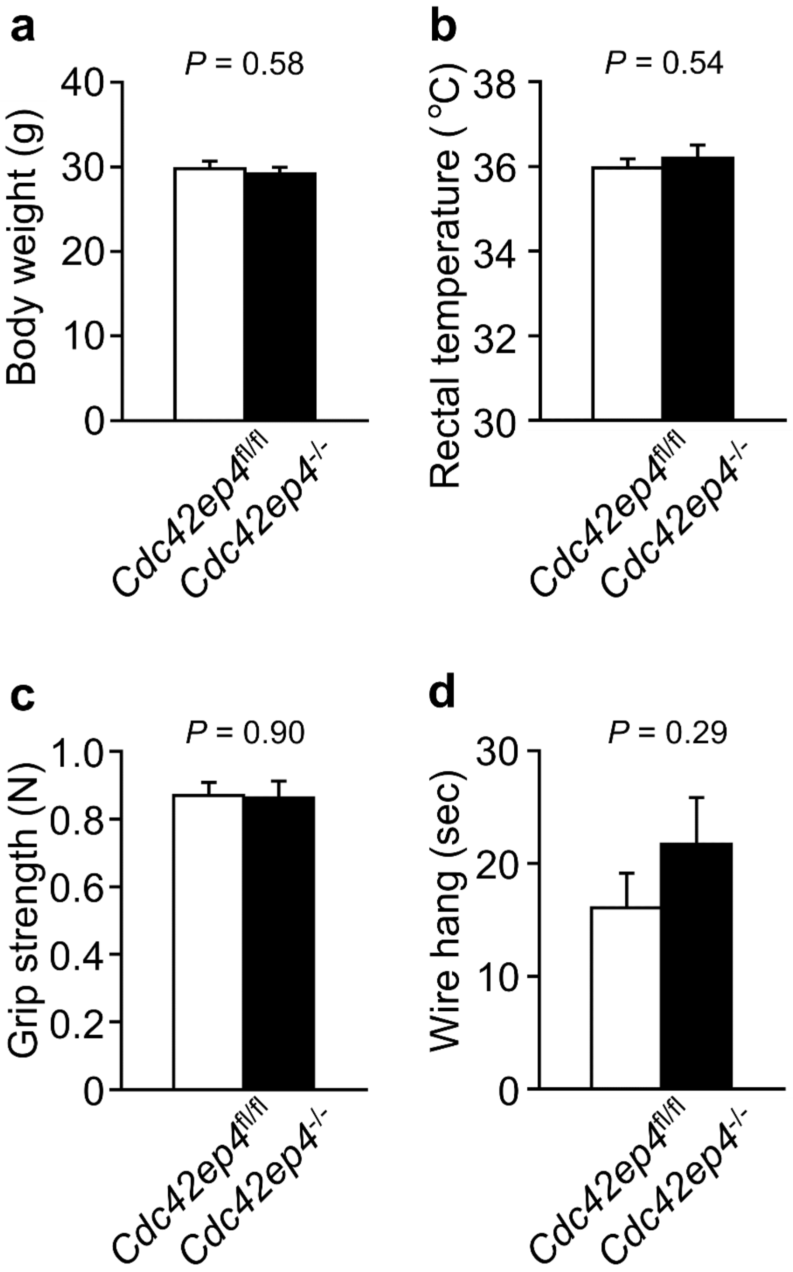

Supplementary Figure 1. Body weight, rectal temperature, and the muscle strength tests

(**a**) Body weight [$F_{1,24}$=0.32, P=0.58], (**b**) rectal temperature [$F_{1,24}$=0.38, P=0.54], (**c**) grip strength [$F_{1,24}$=0.02, P=0.90], and (**d**) wire hang latency [$F_{1,24}$=1.19, P=0.29] of *Cdc42ep4*$^{fl/fl}$ (WT) and *Cdc42ep4*$^{-/-}$ (KO) mice. (n=13, 13, one-way ANOVA.) Data represented as mean±s.e.m.

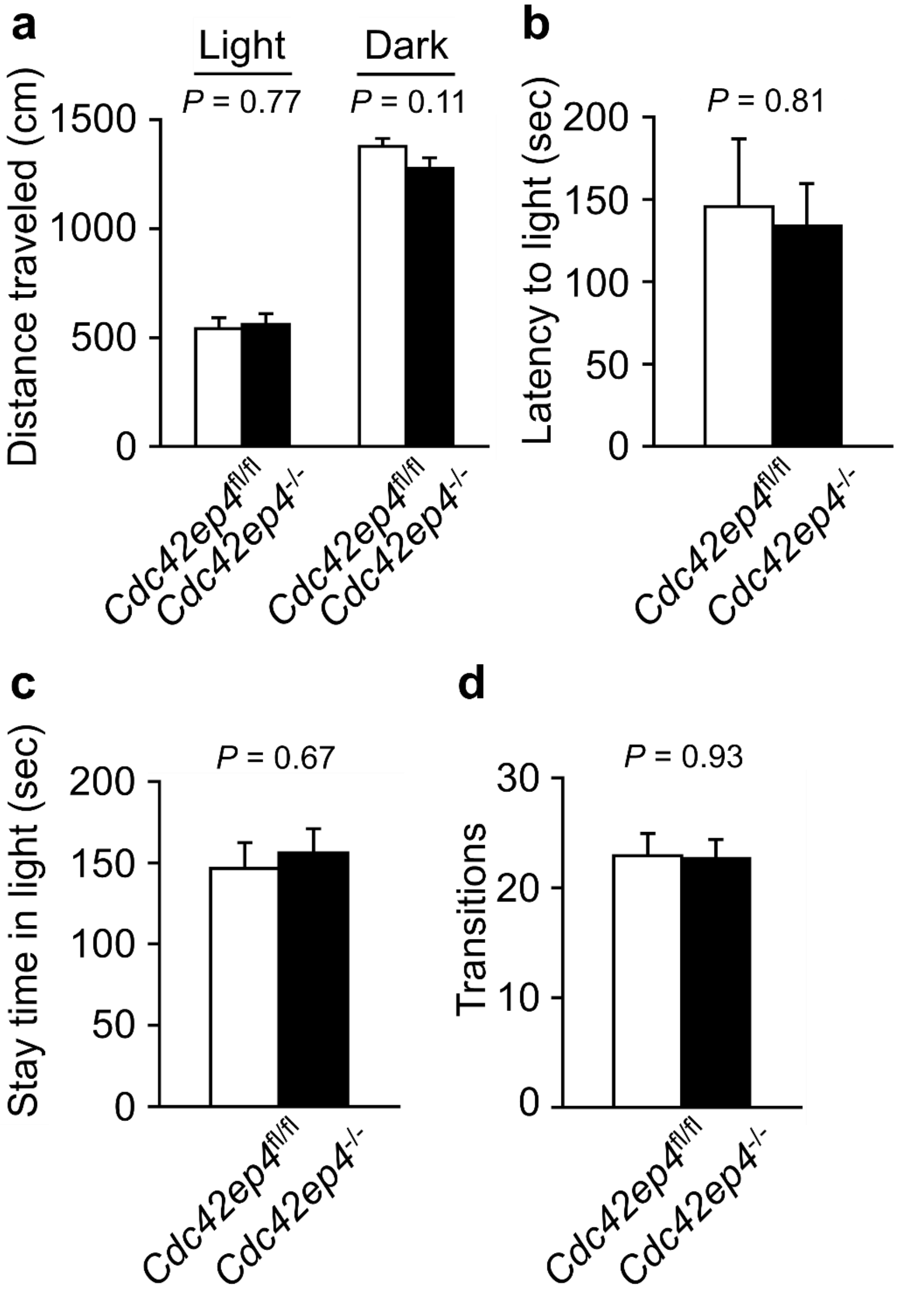

Supplementary Figure 2. The light/dark transition test

(**a**) Distance traveled in the light [$F_{1,24}$=0.09, *P*=0.77] and dark chambers [$F_{1,24}$=2.70, *P*=0.11], (**b**) latency until the first entry into the light chamber [$F_{1,24}$=0.06, *P*=0.81], (**c**) time spent in the light chamber [$F_{1,24}$=0.19, *P*=0.67], and (**d**) number of transitions across the light/dark border [$F_{1,24}$=0.01, *P*=0.93] of WT and KO mice. (*n*=13, 13, one-way ANOVA.) Data represented as mean±s.e.m.

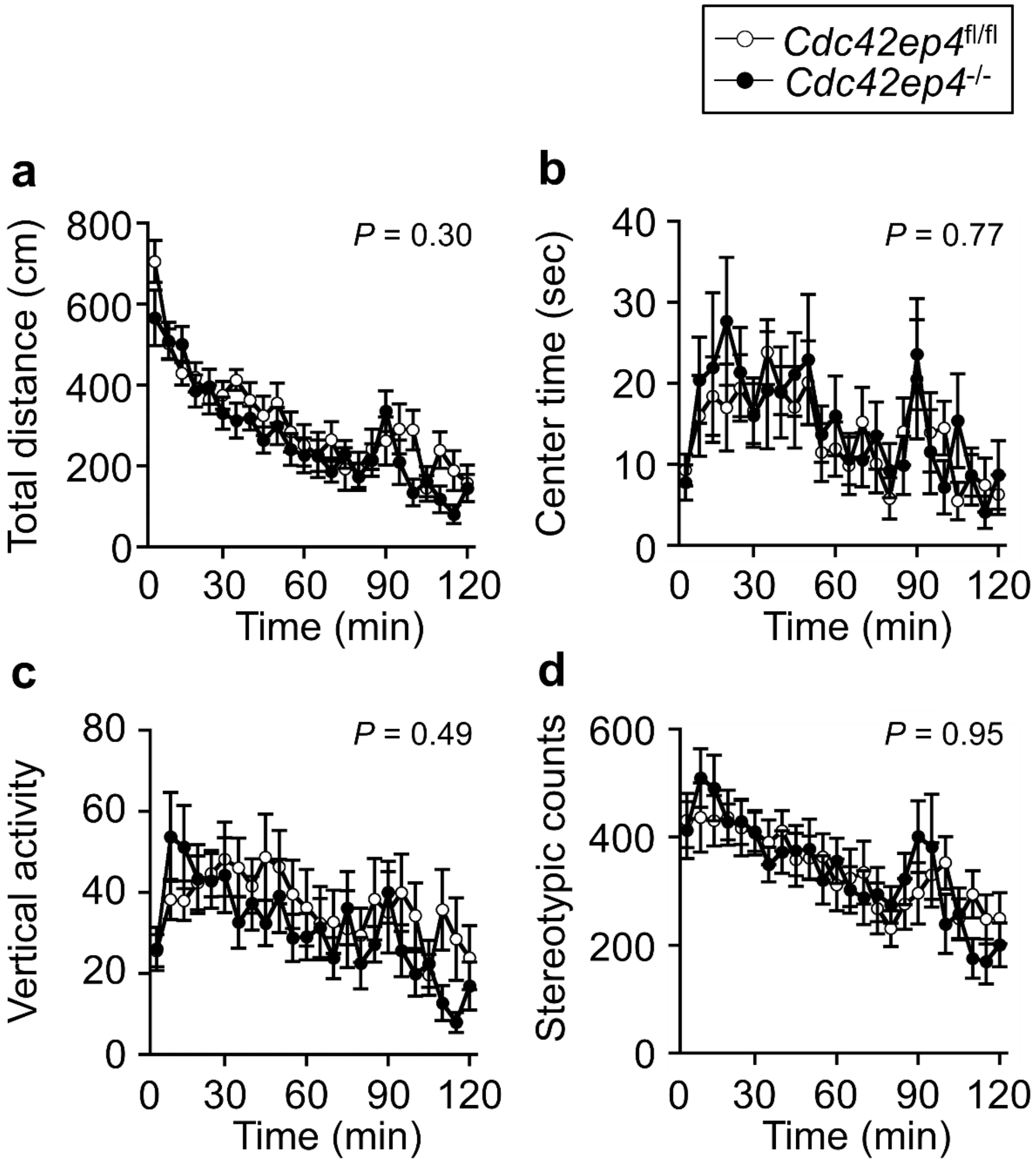

Supplementary Figure 3. The open field test

(**a**) The total distance traveled in the first 120 min after entry into a novel light chamber [$F_{1,24}$=1.11, P=0.30, genotype × time interaction, $F_{23,552}$=1.45, P=0.08], (**b**) time spent near the center of the chamber [$F_{1,24}$=0.09, P=0.77, genotype x time interaction, $F_{23,552}$=0.65, P=0.90], (**c**) frequency of rearing events [$F_{1,24}$=0.49, P=0.49, genotype x time interaction, $F_{23,552}$=1.59, P=0.04], and (**d**) stereotypic movements [$F_{1,24}$=0.003, P=0.95, genotype × time interaction, $F_{23,552}$=1.25, P=0.20] of WT and KO mice. (n=13, 13, two-way repeated measures ANOVA.) Data represented as mean ±s.e.m.

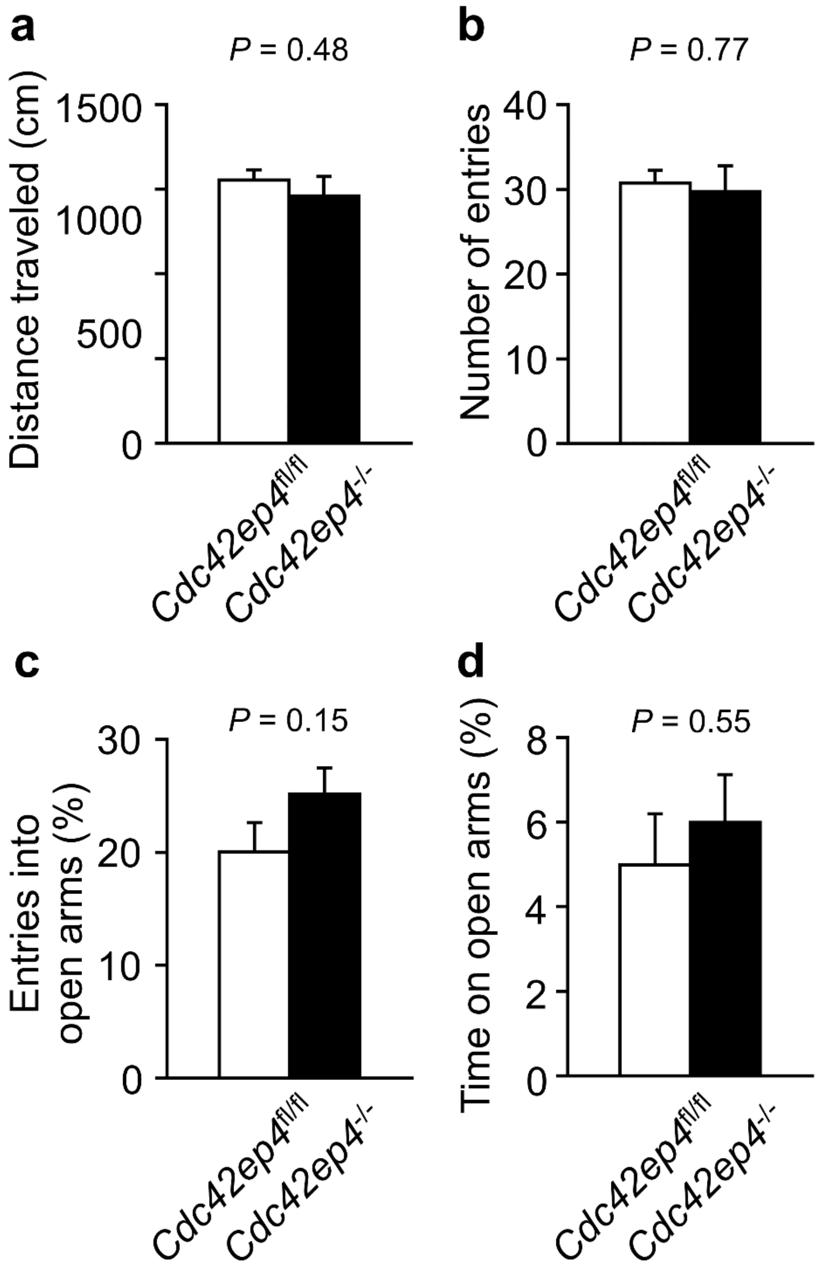

Supplementary Figure 4. The elevated plus maze test

(**a**) Distance traveled [$F_{1,24}$=0.51, P=0.48], (**b**) total number of entries into open and closed arms [$F_{1,24}$=0.09, P=0.77], (**c**) percentage of entries into open arms [$F_{1,24}$=2.22, P=0.15], and (**d**) percentage of stay time on open arms [$F_{1,24}$=0.37, P=0.55] of WT and KO mice. (n=13, 13, one-way ANOVA.) Data represented as mean±s.e.m.

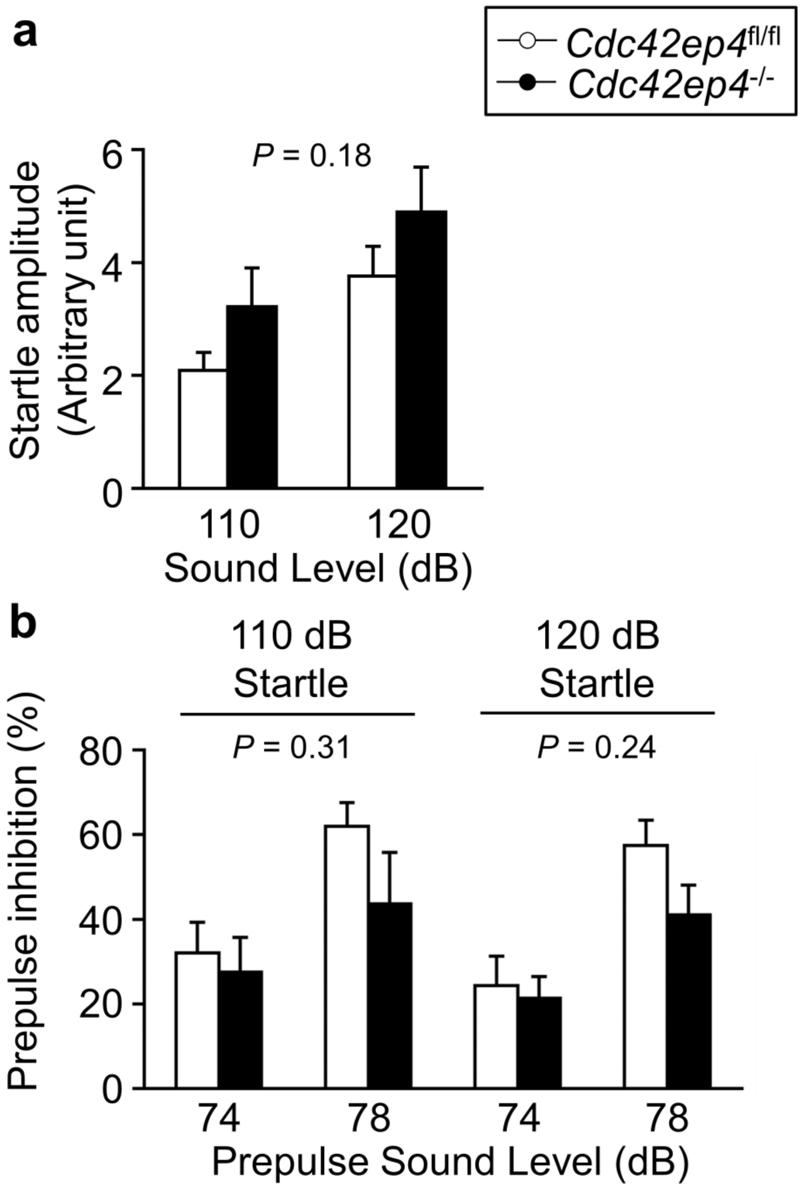

Supplementary Figure 5. The acoustic startle test and prepulse inhibition test

(**a**) Startle amplitude (arbitrary unit) against acoustic stimuli of two distinct loudness (110 dB or 120 dB)[$F_{1,24}$=1.95, P=0.18, genotype × sound interaction, $F_{1,24}$=0.0001, P=0.99], and (**b**) percent reduction of startle amplitude in the presence of a preceding acoustic stimulus (prepulse of 110 dB or 120 dB) [110 dB; $F_{1,24}$=1.09, P=0.31, genotype x sound interaction, $F_{1,24}$=1.55, P=0.23. 120 dB; $F_{1,24}$=1.45, P=0.24, genotype × sound interaction, $F_{1,24}$=2.97, P=0.10] of WT and KO mice. (n=13, 13, two-way repeated measures ANOVA.) Data represented as mean±s.e.m.

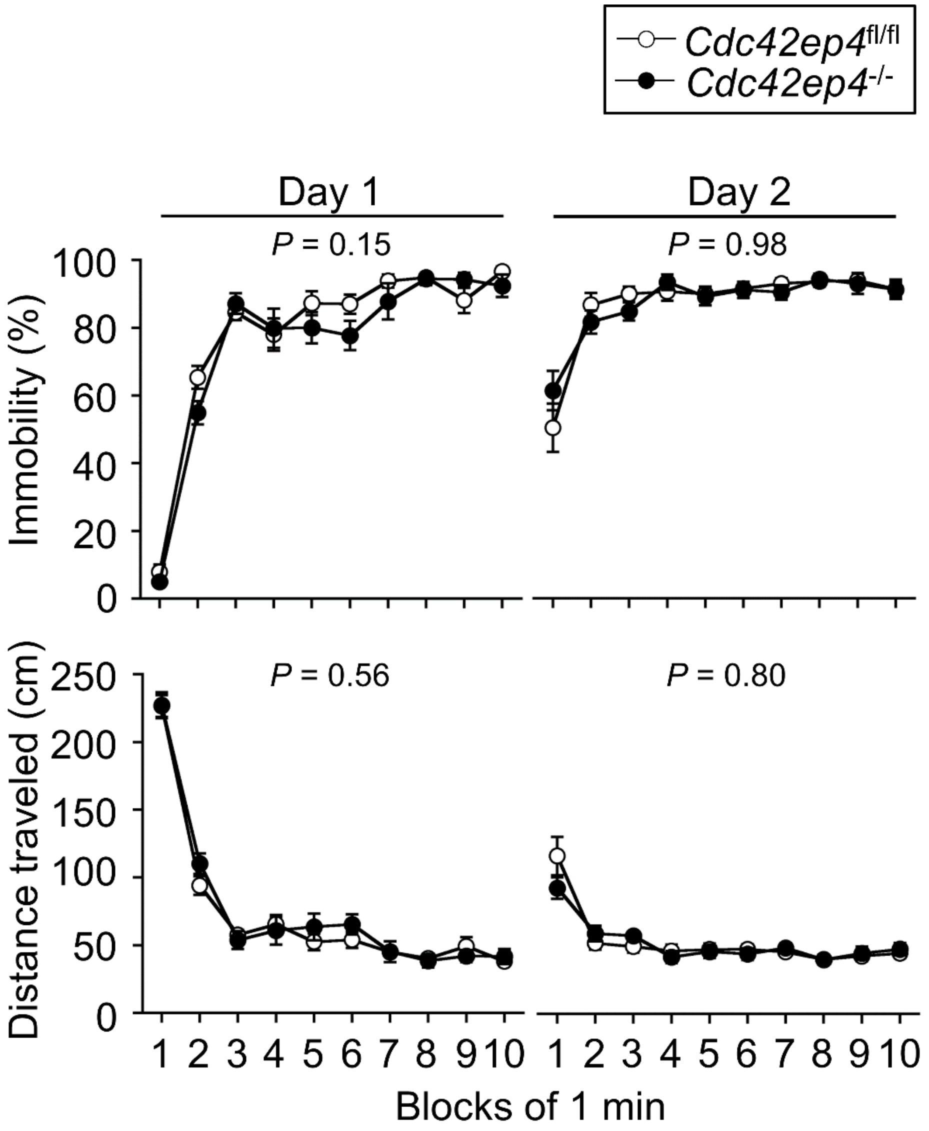

Supplementary Figure 6. Porsolt forced swim test

(top) Percent immobility [Day 1; $F_{1,24}$=2.23, P=0.15, genotype × block interaction, $F_{9,216}$=1.42, P=0.18, Day 2; $F_{1,24}$=0.001, P=0.98, genotype × block interaction, $F_{9,216}$=1.24, P=0.27], and (bottom) distance traveled in water [Day 1; $F_{1,24}$=0.35, P=0.56, genotype × block interaction, $F_{9,216}$=0.78, P=0.64, Day 2; $F_{1,24}$=0.07, P=0.80, genotype × block interaction, $F_{9,216}$=1.71, P=0.09] of WT and KO mice. (n=13, 13, two-way repeated measures ANOVA.) Data represented as mean±s.e.m.

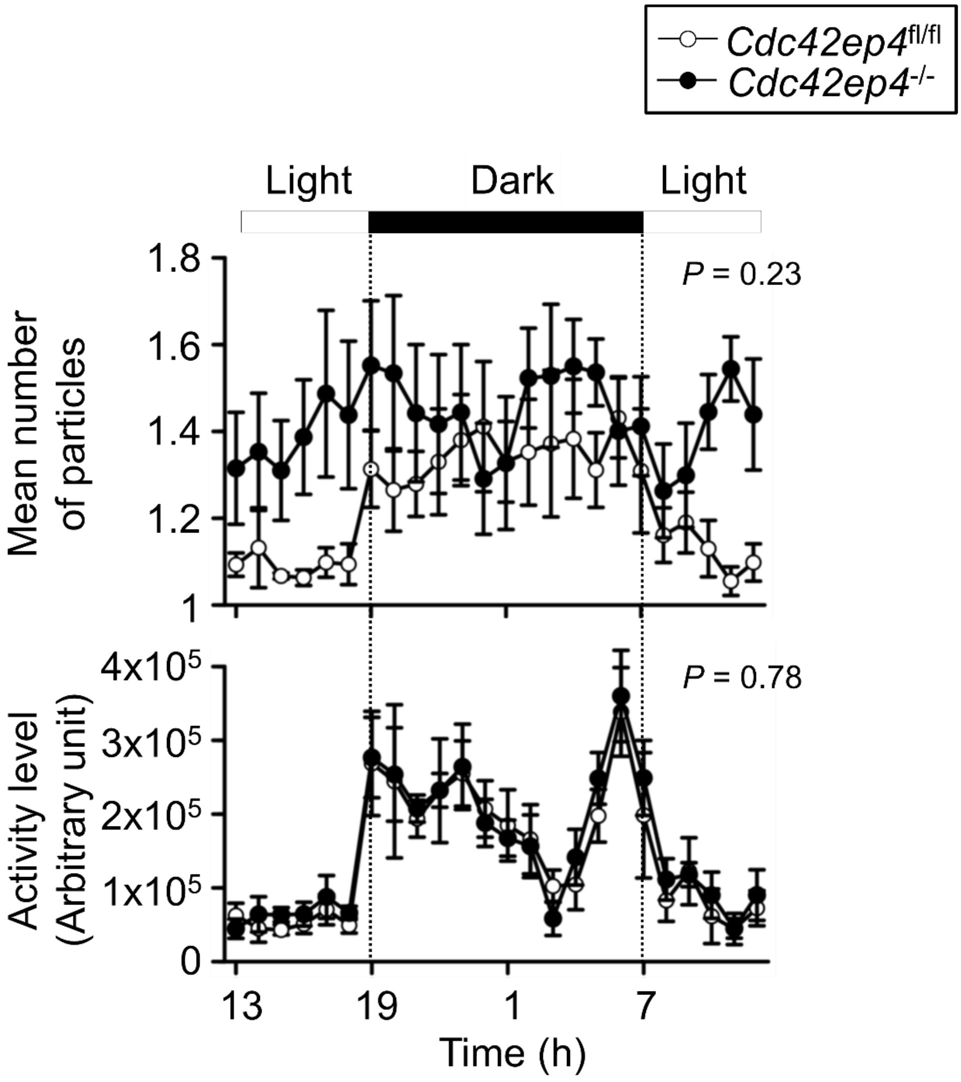

Supplementary Figure 7. Diurnal social interaction and locomotor activity monitoring in the home cage

(top) Diurnal oscillation of the social interaction [$F_{1,8}$=1.72, P=0.23, genotype × time interaction, $F_{23,184}$=1.59, P=0.05] and (bottom) locomotor activity in the home cage through the light/dark phases [$F_{1,8}$=0.09, P=0.78, genotype × time interaction, $F_{23,184}$=0.23, P=1.00] of WT and KO mice. (n=5, 5 pairs, two-way repeated measures ANOVA.) Data represented as mean±s.e.m.

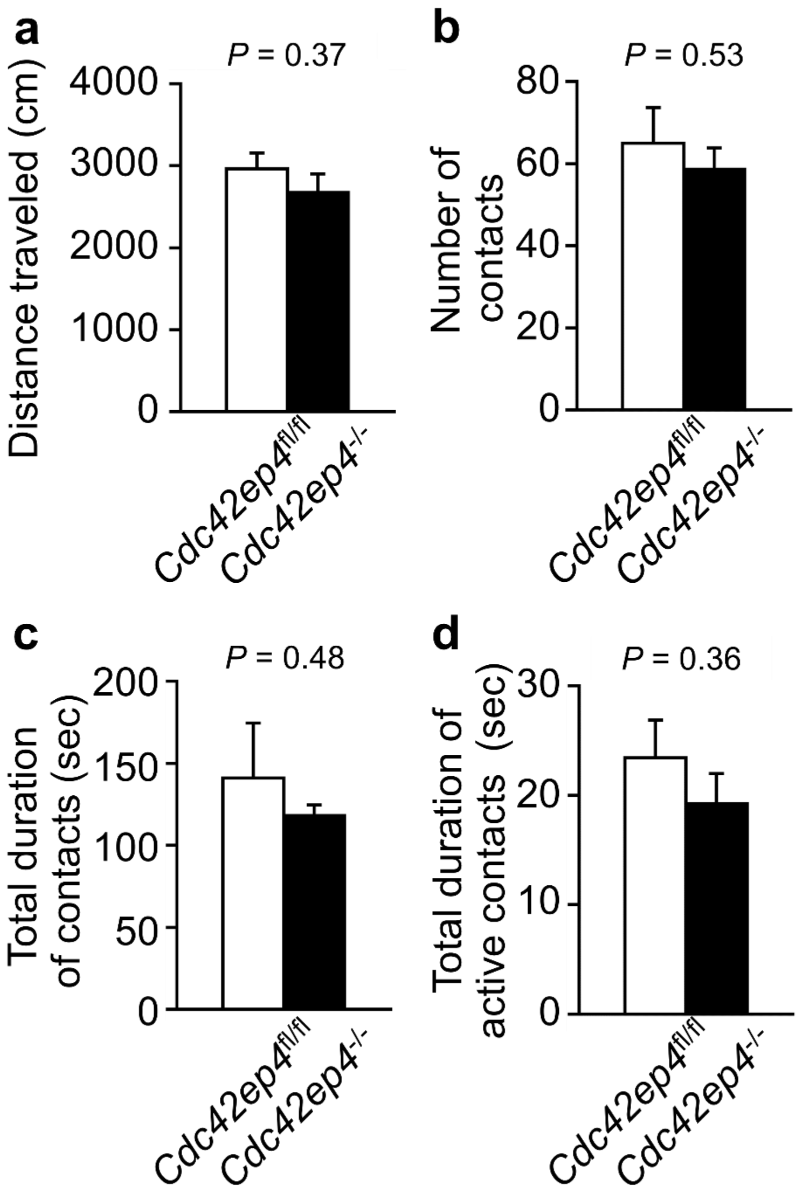

Supplementary Figure 8. The social interaction test (single chamber)

(**a**) Distance traveled [$F_{1,9}$=0.90, P=0.37], (**b**) total number of contacts [$F_{1,9}$=0.43, P=0.53], (**c**) total duration of contacts [$F_{1,9}$=0.54, P=0.48], and (**d**) total duration of active contacts [$F_{1,9}$=0.94, P=0.36] of WT and KO mice. (n=5, 6 pairs, one-way ANOVA.) Data represented as mean±s.e.m.

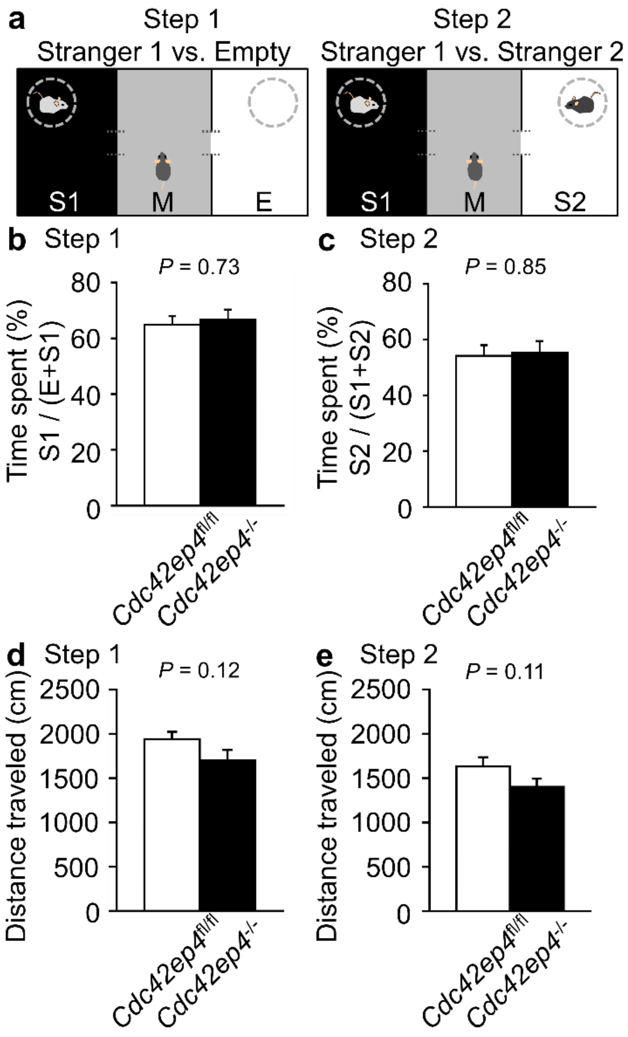

Supplementary Figure 9. The social interaction test (three chambers)

(**a**) Schematic diagrams of the three-chamber sociability and social novelty preference tests. Setup for the sociability test (Step1). The time spent in the middle habituated chamber (M), in the left chamber containing an unfamiliar C57BL/6J mouse (Stranger 1, S1) in a wire cage, and in the right chamber with an empty wire cage (**e**) were separately measured. (right) The social novelty preference test (Step2) was conducted likewise, except for a novel unfamiliar C57BL/6J mouse (Stranger 2, S2) caged in the right chamber in addition to the now-familiar S1 mouse remaining in the left cage/chamber. (**b**) Time spent (Step1) [$F_{1,24}$=0.12, P=0.73], (**c**) Time spent (Step2) [$F_{1,24}$=0.04, P=0.85], (**d**) Distance traveled (Step1) [$F_{1,24}$=2.65, P=0.12], and (**e**) Distance traveled (Step2) [$F_{1,24}$=2.78, P=0.11] of WT and KO mice. (n=13, 13, one-way ANOVA.) Data represented as mean±s.e.m.

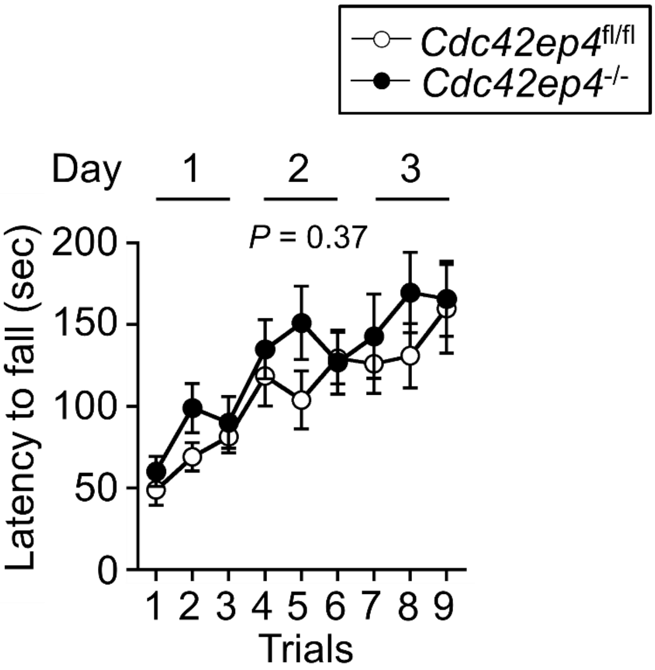

Supplementary Figure 10. The rotating rod (Rota-rod) test

The duration in which WT and KO mice (n=13, 13) kept pace with a rotating rod with a constant acceleration (4—40 rpm over 5 min) increased through 6 trials in three days [$F_{1,24}$=0.82, P=0.37, genotype × trial interaction, $F_{8,192}$=0.99, P=0.44 by two-way repeated measures ANOVA]. Data represented as mean±s.e.m.

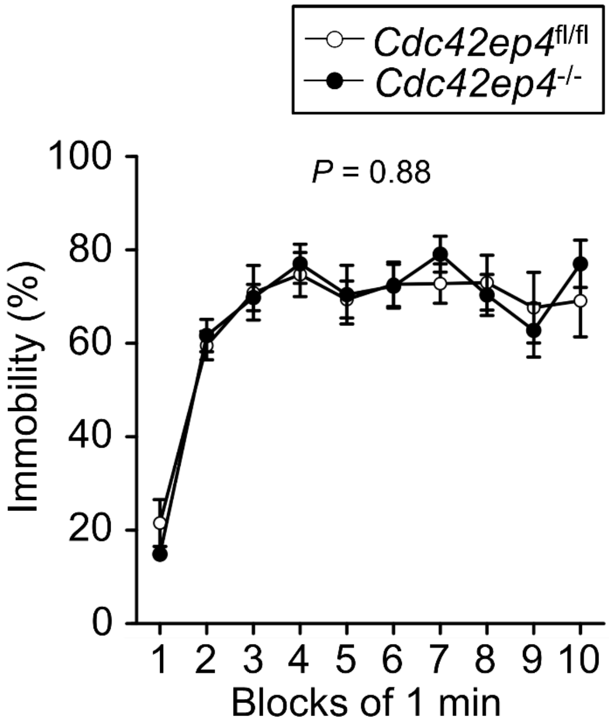

Supplementary Figure 11. The tail suspension test

Comparable percent immobility of WT and KO mice (*n*=12, 12) suspended in the tail. [$F_{1,22}$=0.025, *P*=0.88, genotype × block interaction, $F_{9,198}$=0.44, *P*=0.91 by two-way repeated measures ANOVA.] Data represented as mean±s.e.m.

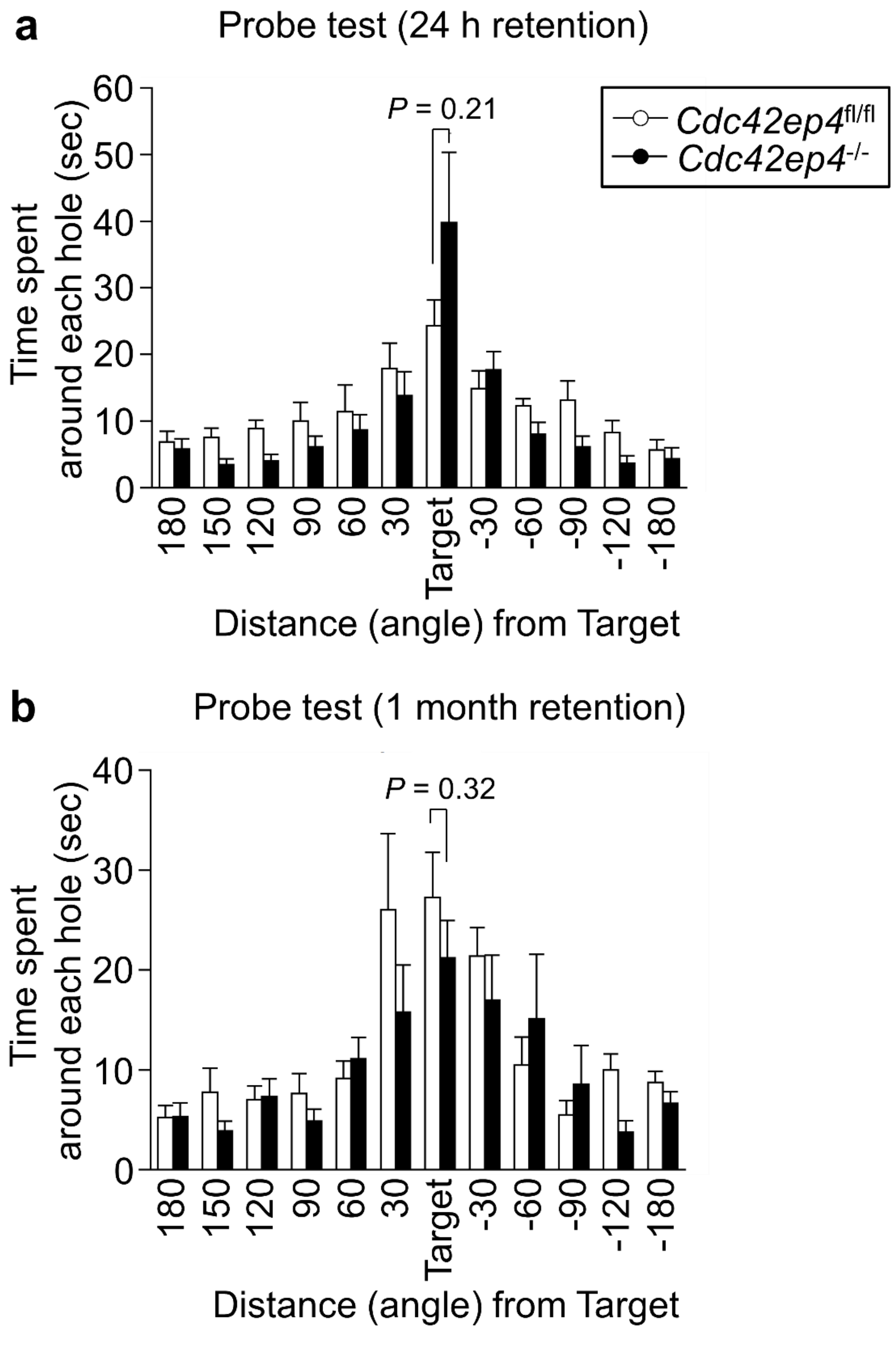

Supplementary Figure 12. Barnes maze test

(**a**, **b**) Probe test results showing the time spent near the escape hole (target) at 24 h (**a**) and 1 month (**b**) after the training session. [24 h; $F_{1,15}$=1.73, P=0.21, 1 month; $F_{1,15}$=1.08, P=0.32] of WT and KO mice. (n=8, 9, one-way ANOVA.) Data represented as mean±s.e.m.

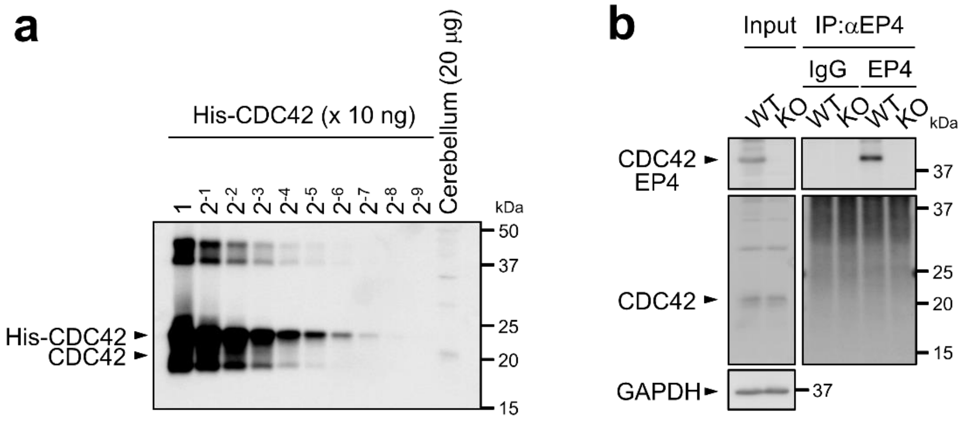

Supplementary Figure 13. Assessment of endogenous CDC42 in the mouse cerebellar lysate

(**a**) The content of endogenous CDC42 in the mouse cerebellar lysate was estimated. The lysate was supplemented with recombinant human CDC42 protein, serially diluted, and immunoblotted for CDC42.

(**b**) Co-immunoprecipitaion/immunoblot (IP/IB) assay of CDC42EP4 with CDC42 from WT and KO cerebellar lysate. (Input) IB for CDC42EP4, CDC42, and GAPDH detected each protein except for CDC42EP4 in KO. (IP) Anti-CDC42EP4 antibody (αEP4), but not random IgG (IgG), pulled down CDC42EP4 from WT cerebellar lysate, when CDC42 was undetectable.

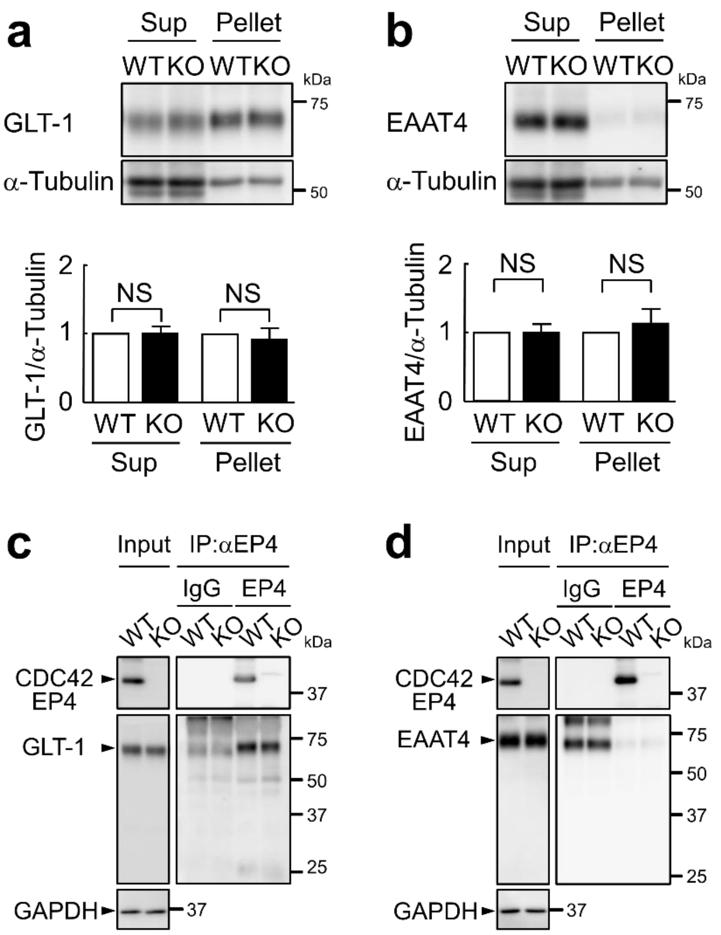

Supplementary Figure 14. Biochemical assessment of two other glutamate transporters

(**a**, **b**) Pellet/supernatant assay results on the quantity and extractability of GLT-1/EAAT2, and EAAT4 in WT and KO cerebella. There was no significant difference in their amount and partitioning by genotype. (*n*=3. NS, *P*>0.05 by *t*-test.) The same membranes were reprobed for α-tubulin as a loading control, which was used for normalization. Data represented as mean±s.e.m.

(**c**, **d**) Co-immunoprecipitaion/immunoblot (IP/IB) assay of CDC42EP4 with GLT-1 and EAAT4 from WT and KO cerebellar lysate. The same membranes were reprobed for GAPDH as a loading control. Unlike GLAST, GLT-1 and EAAT4 were not co-immunoprecipitated with CDC42EP4.

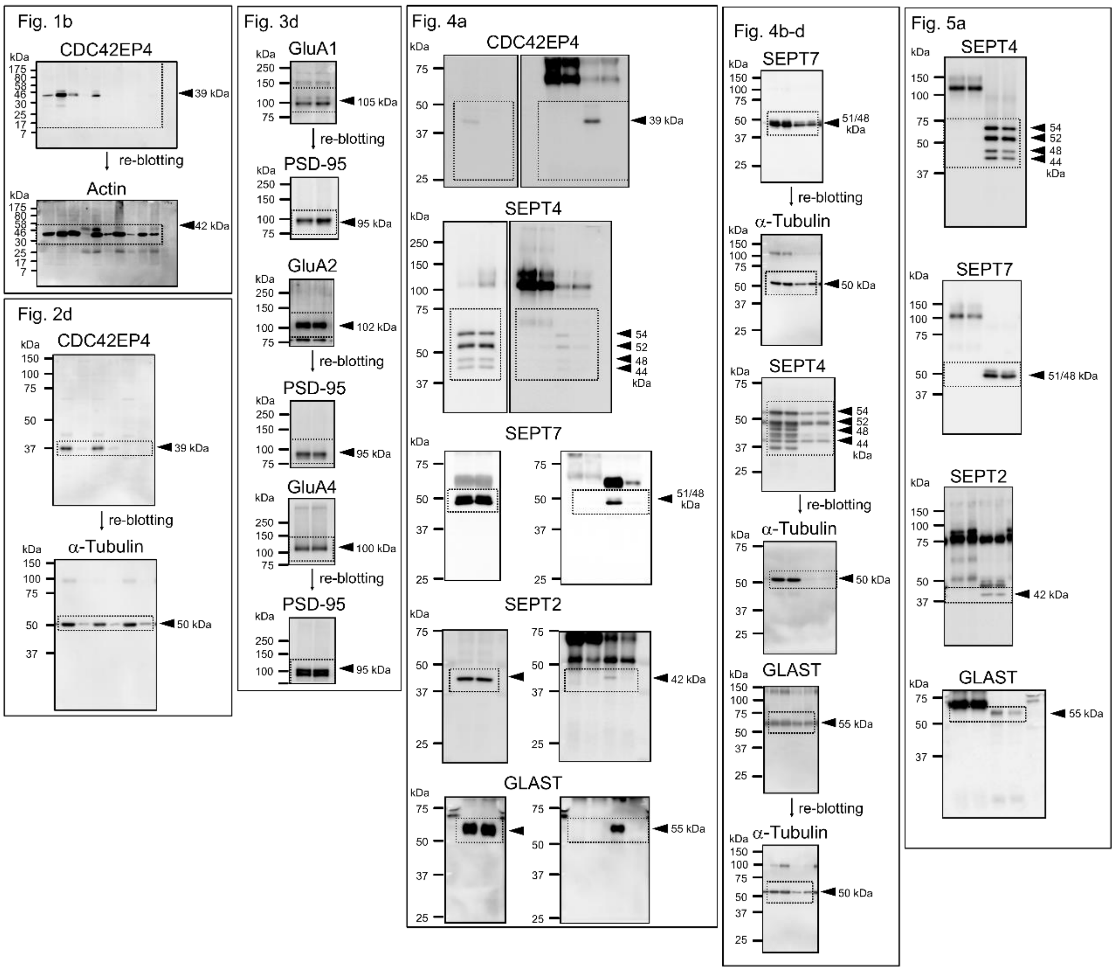

Supplementary Figure 15. Representative entire images of immunoblot

Boxed areas were cropped for designated figures.
